# Productivity losses from short-term work absence due to neoplasms in Poland

**DOI:** 10.1038/s41598-024-53878-4

**Published:** 2024-02-08

**Authors:** Błażej Łyszczarz

**Affiliations:** Department of Health Economics, Nicolaus Copernicus University in Toruń, Sandomierska 16, 85-830 Bydgoszcz, Poland

**Keywords:** Cancer epidemiology, Health care economics, Occupational health, Public health

## Abstract

Previous evidence on productivity losses from neoplasms focuses mostly on the economic burden from mortality, covers single cancer diagnoses and neglects non-malignant neoplasms. This study aims to broaden this perspective by analysing losses resulting from work absence and all neoplasm diagnoses. The analysis applies the human capital method and social insurance data to estimate productivity losses attributable to neoplasm-related short-term work absence in Poland in the period 2012–2022. The productivity losses due to work absence attributable to all neoplasms in Poland were €583 million in 2012 (0.143% of gross domestic product) and they increased to €969 million in 2022 (0.164%). Around 60% of the losses were associated with cancers while the remaining part of the burden was due to non-malignant neoplasms. The neoplasms that led to the highest losses were benign neoplasms, breast cancer, colorectum cancer and prostate cancer. The cancer sites characterised by the greatest losses per absence episode were brain cancer, lung cancer and oesophageal cancer. For most of the neoplasms, we observed increasing losses in an 11-year period analysed. Investing in effective public health policies that tackle neoplasms has the potential to reduce both the health burden and economic losses resulting from these diseases.

## Introduction

Cancer is a major health concern worldwide and the burden of this disease incidence and mortality is growing rapidly^1^. This translates to severe economic consequences in terms of healthcare spending aimed at cancer treatment (direct costs)^2^ and productivity losses resulting from the inability to work of those affected (indirect costs)^3^. The former category of direct costs is conceptually intuitive as it encompasses expenditures associated with producing and delivering health services aimed at disease treatment. On the other hand, indirect costs are a more abstract concept because they do not result in financial flows per se, they rather illustrate potential losses that society bears due to the sick’s inability to perform economic activities^4^.

Numerous studies have scrutinised productivity losses from cancer and these either focused on specific cancer diseases^5–8^ or analysed a range of cancer diagnoses together^9–12^. For the latter category of studies, most investigated a single category of indirect costs—usually mortality losses^9–14^. The focus on the consequences of cancer deaths is not surprising because mortality constitutes a key health-related burden of neoplasms. Yet, cancer morbidity also yields a significant economic burden. A systematic review of breast cancer indirect costs research^3^ lists several studies finding that morbidity losses exceed mortality losses, e.g.^8,15,16^. The economic analysis of Poland’s cancer absenteeism for 2009 shows that sick leave absence constituted ~ 19% of the total indirect costs^17^.

This study aims to shed more light on the economic magnitude of the morbidity burden resulting from neoplasms. This is to be done by analysing time trends in sick leave absence and resulting productivity losses due to all neoplasms (both malignant and non-malignant) in Poland in 11 years of 2012–2022 based on social insurance data. The contributions of this research are as follows. To begin with, a decade-long analysis provides a comprehensive picture of losses evolution over time; this approach is crucial to understanding the dynamics of losses because we experience a growing incidence of cancer and this plausibly translates to an increasing economic burden. Secondly, this study uses work absence data on every malignant-related diagnosis as defined by the International Statistical Classification of Diseases and Related Health Problems, 10^th^ Revision (ICD-10). Until now, the economic studies of neoplasm burden, either focused on single cancers^5,6^ or on aggregated groups of diseases classified by cancer site^11^ or did not account for non-malignant neoplasms^9,10^. Using a complete list of neoplasm diagnoses (ICD-10 codes: C00-D48), this study aims to provide a more comprehensive picture of the economic burden of neoplasms. Clearly, absenteeism is only one indirect cost category and this approach does not assess the burden of disease due to presenteeism or mortality; yet, it provides additional insight into the topic. Thirdly, the study additionally uses data aggregated by the cancer site and this allows us to compare sick leave absence losses with mortality losses in a single country (Poland). This was feasible because I used the same classification of cancers by site as reported previously in Ortega-Ortega et al.^11^. With this comparison, we could test whether in Poland the cancers that lead to high mortality losses are the ones that also account for the greatest work absence losses. This complements the picture of neoplasm-related losses in Poland and formulates the direction of future research in other countries' settings. Finally, social insurance data (which is a basis for empirical analysis here) in Poland is published with a short time lag and this allows for an up-to-date investigation. With this advantage over other epidemiological data which is made available with much longer lags (e.g. mortality), I was able to analyse the impact of the three years of the COVID-19 pandemic on both absence and productivity losses.

## Material and methods

The study used the human capital method (HCM), societal perspective and social insurance data to estimate productivity losses (indirect costs) attributable to neoplasm-related short-term work absence in Poland in an 11-year period of 2012–2022. Using HCM in a morbidity-based analysis, such as in this study, means that indirect costs are estimated as the value of lost productive time due to acute illness and/or short- and long-term disabilities^3,18,19^. In other words, the output that is not produced because of a sick person’s work absence is considered to be an economic loss and the sum of a potential output unproduced by all the sick approximates the social loss resulting from a disease.

The scope of this study encompasses short-term (sick leave) absence solely; long-lasting (> 180 days) and permanent inability to work are not accounted for here. Short-term absence is identified based on the number and duration of sickness leave certificates issued by physicians to the sick insured. This data is routinely reported to the Social Insurance Institution (SII) which provides social allowances to above 90% of the insured population in Poland (excluding farmers, uniformed services and justice services employees). Short-term absence, as defined here, does not account for absence episodes lasting above 180 days; these are subject to rehabilitation and disability benefits and are not analysed here.

The study investigated the indirect costs of sick leave absence resulting from all neoplasm diseases as defined by the ICD-10 classification. For each of the 88 cancer (malignant) ICD-10 codes (C00-C97) and 48 non-malignant neoplasm ICD-10 codes (D00-D48), I obtained the number of absence days and certificates from the SII’s online database^20^. The single ICD-10 codes were also grouped. For malignant neoplasms, I used the following classification of 23 cancer sites as used by Ortega-Ortega et al. (2021)^11^: oral cavity and pharynx (ICD-10: C00-C14), oesophagus (C15), stomach (C16), colorectum (C18-C21), liver (C22), gallbladder (C23-C24), pancreas (C25), larynx (C32), lung (C33-C34), melanoma skin (C43), breast (C50), cervix uteri (C53), corpus uteri (C54), ovary (C56), prostate (C61), kidney (C64-C65), bladder (C67), brain and central nervous system (C70-C72), thyroid (C73), Hodgkin lymphoma (C81), non-Hodgkin lymphoma (C82-C85, C96), multiple myeloma (C88-C90), and leukaemia (C91-C95). The above groups accounted for 91.5% of all cancer-related absence days throughout the period analysed. For non-malignant neoplasms, three groups were distinguished, namely: in situ neoplasms (D00-D09), benign neoplasms (D10-D36), and neoplasms of uncertain or unknown behaviour (D37-D48).

An indirect cost of a neoplasm-related absence day was calculated as the average daily productivity of a working person. To obtain this, the following socioeconomic measures were used: gross domestic product, number of working days per year, full-time and part-time working population, and age-group-specific average remuneration. For a specific year, the total economy’s GDP was divided by the working population and the number of average working days (accounting for statutory paid holidays). To reflect that productivity changes with age, I adjusted the average per worker GDP with age differences in remuneration. Additionally, because marginal productivity is preferred over average productivity in indirect costs estimation^18^, per worker GDP was adjusted for decreasing marginal productivity by applying a 0.65 coefficient^21–23^. This adjustment is required as production output depends not only on human capital but also on other inputs such as capital or natural resources. To reflect this, marginal productivity was proxied by a 0.65 coefficient which reflects the output elasticity of labour in the Cobb–Douglas production function as used in the European context^24^. For a particular neoplasm or group of neoplasms, a product of absence days and adjusted per worker daily GDP yields a productivity loss.

All the monetary measures are expressed in real terms using 2020 as a base year and deflated using a consumer price index and using Euro (€) currency calculated with the average yearly exchange rates for the whole period (2012–2022)—4.33 PLN per €. No discounting was used as no losses span to future years.

The model parameters and their values for selected years are tabulated in Table 1.

**Table 1:** Summary of socioeconomic parameters of the model for estimating productivity losses associated with neoplasms in Poland in the years 2012–2022.

Parameter (unit)		Parameter value		
	2012	2017	2022	Average 2012–2022
Population (number)	38,533,299	38,433,558	37,766,327	38,306,152
Gross domestic product (€ million)^a^	372,448.4	457,909.5	710,913.1	483,721.4
Exchange rate (PLN per €)	4.19	4.26	4.69	4.33
Consumer price index^b^	103.7	102.0	114.4	102.9
Employment (thousands of working people)				
- full-time employment	14,356	15,226	15,671	15,069
- part-time employment	1,235	1,197	1,072	1,160
Real per worker GDP^a,c^ (€)	27,180	30,677	36,483	30,961
Working days per year (number)^d^	225	224	224	224

PLN—Polish currency (złoty); GDP—gross domestic product. a—values in Euro currency (€) calculated using constant average 2012–2022 exchange rate: 4.33 PLN per €; b—consumer price index is used as a deflator to calculate real GDP; c—for this measure, part-time employment translates to half of the full-time employment; d—the measure accounts for statutory holidays and an average 27 days a year of paid holidays^25^.

## Results

### Neoplasm-related short-term work absence characteristics

Table 2 presents the data on absence characteristics for 23 malignant neoplasm groups classified by cancer site and three non-malignant neoplasm groups; the data refers to the first year (2012), the middle year (2017) and the last year (2022) of the period analysed. More detailed data disaggregated by 88 cancer ICD-10 codes (C00-C97) and 48 non-malignant neoplasm ICD-10 codes (D00-D48) and for each year of the 2012–2022 are shown in Supplementary Table 1 and Supplementary Table 2.

**Table 2:** Neoplasm-related short-term work absence characteristics by cancer site in Poland in the years 2012, 2017 and 2022.

	Number of absence days			Average length of an absence episode (days)			Number of absence days per 1,000 working population		
	2012	2017	2022	2012	2017	2022	2012	2017	2022
oral cavity and pharynx (C00-C14)	133,209	154,310	139,697	26.5	25.5	23.6	8.5	9.4	8.3
oesophagus (C15)	35,146	43,560	41,179	25.2	26.9	24.2	2.3	2.7	2.5
stomach (C16)	136,445	145,211	138,440	26.9	25.7	21.8	8.8	8.8	8.3
colorectum (C18-C21)	446,955	564,747	564,655	24.9	24.3	21.5	28.7	34.4	33.7
liver (C22)	25,206	30,723	33,165	22.9	23.9	21.7	1.6	1.9	2.0
gallbladder (C23-C24)	22,258	25,342	22,465	25.0	23.2	21.6	1.4	1.5	1.3
pancreas (C25)	72,732	85,303	84,117	25.7	25.1	21.9	4.7	5.2	5.0
larynx (C32)	77,697	69,560	44,543	28.3	26.3	23.8	5.0	4.2	2.7
lung (C33-C34)	440,024	427,084	323,990	26.1	26.8	24.5	28.2	26.0	19.4
melanoma skin (C43)	77,413	101,548	100,181	20.3	18.5	14.4	5.0	6.2	6.0
breast (C50)	942,255	1,129,149	1,313,504	24.7	23.3	20.0	60.4	68.8	78.5
cervix uteri (C53)	138,187	124,693	107,996	26.7	24.9	22.9	8.9	7.6	6.5
corpus uteri (C54)	123,902	155,102	160,723	25.5	24.1	23.1	7.9	9.4	9.6
ovary (C56)	185,182	197,921	189,449	23.0	22.5	20.8	11.9	12.1	11.3
prostate (C61)	182,757	375,534	449,985	24.6	25.3	23.7	11.7	22.9	26.9
kidney (C64-65)	149,074	160,119	142,100	24.9	24.4	22.5	9.6	9.7	8.5
bladder (C67)	143,353	169,287	153,284	19.1	19.3	17.0	9.2	10.3	9.2
brain and central nervous system (C70-72)	145,093	170,952	157,201	29.0	30.6	28.7	9.3	10.4	9.4
thyroid (C73)	123,683	200,221	201,954	19.0	19.1	16.9	7.9	12.2	12.1
Hodgkin lymphoma (C81)	61,489	64,723	62,675	21.1	20.8	17.8	3.9	3.9	3.7
non-Hodgkin lymphoma (C82-C85, C96)	114,573	150,167	142,370	22.2	22.4	19.5	7.3	9.1	8.5
multiple myeloma (C88, C90)	42,587	63,292	57,206	23.2	24.4	20.1	2.7	3.9	3.4
leukaemia (C91-C95)	119,289	138,188	108,847	22.3	21.9	17.1	7.7	8.4	6.5
in situ neoplasms (D00-D09)	24,706	38,786	65,173	17.4	17.6	18.2	1.6	2.4	3.9
benign neoplasms (D10-D36)	2,207,705	2,303,394	2,182,676	18.5	17.2	15.4	141.6	140.3	130.4
neoplasms of uncertain or unknown behaviour (D37-D48)	669,243	1,094,116	1,285,551	17.3	16.6	14.6	42.9	66.6	76.8
all cancers (C00-C97)	4,330,588	5,192,758	5,162,595	24.1	23.5	20.7	277.8	316.2	308.3
all neoplasms (C00-D48)	7,232,242	8,629,054	8,695,995	21.3	20.4	18.0	463.9	525.4	519.4
all diseases (A00-Z99)	206,776,323	245,568,567	238,486,559	12.5	12.4	11.0	13,262.5	14,952.7	14,244.0

Data for each year of the 2012–2022 period is available in Supplementary table 1 and Supplementary table 2.

There were over 7.2 million work absence days related to all neoplasms combined in 2012 in Poland and this number increased to 8.6–8.7 million in 2017 and 2022. Of this number, approx. 60% of workdays lost were associated with malignant neoplasms (C00-C97) and this share was stable across the period, with a slight increase to ~ 64% in 2020, the first COVID-19 pandemic year. Cancer-related (neoplasm-related) absence constituted 2.1–2.2% (3.2–3.7%) of all-cause absence days which ranged from 207 million (2012) to 256 million (2020) across the period.

Considering work absence by neoplasm type and site, the highest number of workdays lost was attributed to benign neoplasms (D10-D36) with 2.2–2.3 million days lost throughout the period in most of the years. Breast cancer was the most prevalent cause of absence among site-specific cancers and a notable (39%) increase of days lost was observed for the period analysed for this disease. The other malignant neoplasms characterised by the high work absence were colorectum cancer, lung cancer, and prostate cancer. There were two benign neoplasms which resulted in a high number of absence days and these were leiomyoma of uterus (D25) and benign neoplasm of ovary (D27). The former diagnosis resulted in more absence days than breast cancer in 2012 (963 thousand vs. 942 thousand) but in the following years this number declined and in 2022 it was the second most often reason for sick leave absence in Poland. Considering the benign neoplasm of ovary, its magnitude in terms of work days lost was similar to that of lung cancer (see Supplementary Table 1 for details).

The number of days lost from breast, colorectum and prostate cancer increased throughout the period by 39% (942,255 in 2012 to 1,313,504 in 2022), 26% (446,955 to 564,655) and 146% (182,757 to 449,985), respectively, while for lung cancer, the absence measure declined by 26% (440,024 to 323,990). Generally, for a majority of analysed neoplasm groups, the number of days lost increased from 2012 to 2022 and this growth was the highest for in-situ neoplasms (164%), prostate cancer (146%); neoplasms of uncertain or unknown behaviour (92%); and thyroid cancer (63%). On the other hand, there were five groups of neoplasm with a decline of absence days in the period analysed; these were: larynx (-42.7%); lung (-26.4%); cervix uteri (-21.8%); kidney (-4.7%), and benign neoplasms (-1.1%).

An average length of absence (ALOA) episode was notably higher in all cancer (20.7 days in 2022) and all neoplasm (18 days) groups than for all the diseases combined (11 days). From 2012 to 2022, the ALOA episode declined by 3.4 days in all cancers (-14.1%), 3.3 days in all neoplasms (-15.5%) and 1.5 days in all-cause absence (-11.9%). The declining pattern of absence duration was evident in almost all analysed disease groups; the highest ALOA decline was identified for melanoma skin cancer (-29.3%); leukaemia (-23.2%); breast cancer (-18.9%) and stomach cancer (-18.7%). The only analysed group with an increased ALOA in the period were in situ neoplasms (+ 4.7%).

The effect of the COVID-19 pandemic on the number of absence days resulting from neoplasms is briefly shown in Supplementary Table 3. For all-cause absence, we observe a 7.2% increase in absence days between 2019 (the pre-pandemic year) and 2020 (the first pandemic year). However, for all neoplasms combined the corresponding number declined by 7%, but for all malignant neoplasms combined, it remained almost unchanged (+ 0.3%). The following pandemic years, 2021 and 2022, resulted in a declining number of all-cause absence days as compared to 2020 (~ 6–7% decrease) and this declining trend was similar in all cancers combined in 2021 and 2022 but in all neoplasms combined only in 2021. Considering particular neoplasm groups, the first pandemic year (2020) resulted in a notable growth in absence days from leukaemia (22.4%), non-Hodgkin lymphoma (19.3%) and Hodgkin lymphoma (10.3%), and it was followed by a decline in the two following years in all three diseases. On the other hand, benign neoplasms and lung cancer were at the top of those groups of neoplasms characterised by the greatest decline of absence days in 2020, with a drop of 22.7% and 11.6%, respectively. Generally, no clear picture arises from the analysis of absence days changes during the pandemic period for particular groups of neoplasms and a detailed analysis of this sub-topic is beyond the scope of this paper.

### Productivity losses from neoplasm-related short-term work absence

Productivity losses resulting from all neoplasms short-term absence were €583.4 million in 2012 and they increased to €803.3 million in 2017 and €969.1 million in 2022 (all the above and following monetary measures are expressed in real values calculated by using 2020 as a base year). Malignant neoplasms accounted for around 60% of these losses (€349.4 million in 2012 and €575.3 million in 2022) while the remaining losses were due to non-malignant neoplasms. Of the losses resulting from the analysed groups of neoplasms, the highest was identified for benign neoplasms (€243.2 million in 2022), breast cancer (€146.4 million), neoplasms of uncertain or unknown behaviour (€143.3 million), and colorectum cancer (€62.9 million). The loss per absence episode was higher for all cancers (€2,311 in 2022) than for all neoplasms (€2,010) and double the loss from all-cause absence (€1,159). The absence due to brain and central nervous system cancer generated by far the highest average indirect cost of €3,202 in 2022, followed by lung cancer, oesophageal cancer, oral cavity and pharynx cancer, larynx cancer, and prostate cancer (all above €2,600 in 2022). On the other hand, the average cost of non-malignant neoplasms was lower (Table 3).

**Table 3:** Productivity losses associated with short-term neoplasm-related work absence by cancer site in Poland in the years 2012, 2017 and 2022.

	Total losses (thousand €)			Per 1,000 population losses (€)			Per absence episode losses (€)		
	2012	2017	2022	2012	2017	2022	2012	2017	2022
oral cavity and pharynx (C00-C14)	10,746	14,365	15,569	278.9	373.8	412.2	2,135.6	2,377.6	2,625.4
oesophagus (C15)	2,835	4,055	4,589	73.6	105.5	121.5	2,036.8	2,506.3	2,693.2
stomach (C16)	11,007	13,518	15,428	285.7	351.7	408.5	2,167.6	2,395.6	2,433.1
colorectum (C18-C21)	36,056	52,575	62,928	935.7	1,367.9	1,666.2	2,012.2	2,261.0	2,400.8
liver (C22)	2,033	2,860	3,696	52.8	74.4	97.9	1,845.2	2,225.8	2,417.3
gallbladder (C23-C24)	1,796	2,359	2,504	46.6	61.4	66.3	2,017.5	2,162.4	2,405.0
pancreas (C25)	5,867	7,941	9,374	152.3	206.6	248.2	2,076.2	2,332.9	2,440.0
larynx (C32)	6,268	6,476	4,964	162.7	168.5	131.4	2,281.7	2,446.4	2,650.3
lung (C33-C34)	35,497	39,759	36,107	921.2	1,034.5	956.1	2,103.9	2,495.9	2,731.2
melanoma skin (C43)	6,245	9,454	11,165	162.1	246.0	295.6	1,638.2	1,724.5	1,599.3
breast (C50)	76,013	105,118	146,383	1,972.7	2,735.0	3,876.0	1,992.8	2,170.7	2,232.2
cervix uteri (C53)	11,148	11,608	12,036	289.3	302.0	318.7	2,153.3	2,322.1	2,550.5
corpus uteri (C54)	9,995	14,439	17,912	259.4	375.7	474.3	2,059.6	2,243.5	2,577.2
ovary (C56)	14,939	18,425	21,113	387.7	479.4	559.0	1,854.4	2,097.8	2,318.1
prostate (C61)	14,743	34,960	50,149	382.6	909.6	1,327.9	1,983.7	2,356.8	2,643.0
kidney (C64-65)	12,026	14,906	15,836	312.1	387.8	419.3	2,011.4	2,271.6	2,512.5
bladder (C67)	11,564	15,760	17,083	300.1	410.1	452.3	1,538.9	1,800.3	1,897.0
brain and central nervous system (C70-72)	11,705	15,915	17,519	303.8	414.1	463.9	2,339.1	2,846.5	3,201.6
thyroid (C73)	9,978	18,639	22,507	258.9	485.0	595.9	1,534.8	1,775.7	1,885.0
Hodgkin lymphoma (C81)	4,960	6,025	6,985	128.7	156.8	184.9	1,700.5	1,936.8	1,980.4
non-Hodgkin lymphoma (C82-C85, C96)	9,243	13,980	15,866	239.9	363.7	420.1	1,789.5	2,081.9	2,169.0
multiple myeloma (C88, C90)	3,436	5,892	6,375	89.2	153.3	168.8	1,869.2	2,274.1	2,243.3
leukaemia (C91-C95)	9,623	12,865	12,130	249.7	334.7	321.2	1,799.1	2,041.7	1,908.2
in situ neoplasms (D00-D09)	1,993	3,611	7,263	51.7	93.9	192.3	1,402.6	1,635.3	2,028.3
benign neoplasms (D10-D36)	178,098	214,433	243,248	4,621.9	5,579.3	6,440.9	1,494.4	1,599.9	1,714.9
neoplasms of uncertain or unknown behaviour (D37-D48)	53,989	101,856	143,268	1,401.1	2,650.2	3,793.5	1,392.0	1,545.9	1,630.8
all cancers (C00-C97)	349,354	483,417	575,345	9,066.3	12,578.0	15,234.4	1,947.2	2,190.5	2,310.6
all neoplasms (C00-D48)	583,434	803,318	969,125	15,141.0	20,901.5	25,661.1	1,722.1	1,899.9	2,009.5
all diseases (A00-Z99)	15,872,897	21,594,093	25,167,396	411,926.8	561,855.2	666,397.8	956.2	1,091.0	1,158.6

Data for each year of the 2012–2022 period is available in Supplementary table 4 and Supplementary table 5.

**Figure 1:**
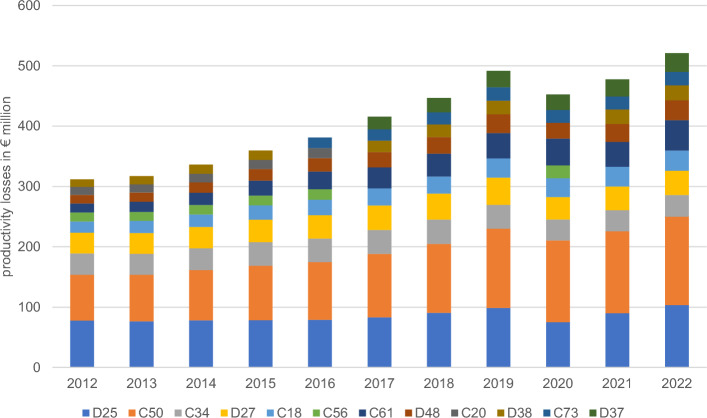
Ten neoplasm diagnoses of highest productivity losses in Poland 2012–2022. Notes: C18—colon cancer; C20—rectal cancer; C34—bronchus and lung cancer; C50—breast cancer; C56—ovarian cancer; C61—prostate cancer; C73—thyroid cancer; D25—leiomyoma of uterus; D27—benign neoplasm of ovary; D37—neoplasm of uncertain or unknown behaviour of oral cavity and digestive organs; D38—neoplasm of uncertain or unknown behaviour of middle ear and respiratory and intrathoracic organs; D48—neoplasm of uncertain or unknown behaviour of other and unspecified sites.

Ten conditions resulting in the highest economically burdening short-term work absence in 2012 were leiomyoma of uterus (ICD-10 code: D25), breast cancer (C50), lung cancer (C34), benign neoplasm of ovary (D27), colon cancer (C18), ovarian cancer (C56), prostate cancer (C61), neoplasm of uncertain or unknown behaviour of other and unspecified sites (D48), rectal cancer (C20) and neoplasm of uncertain or unknown behaviour of middle ear and respiratory and intrathoracic organs (D38). In 2022, the losses from ovarian cancer and rectal cancer became relatively lower and, as a result, thyroid cancer (C73) and neoplasm of uncertain or unknown behaviour of oral cavity and digestive organs (D37) were identified in the top ten economically burdening neoplasm diagnoses (Fig. 1). For each of the years analysed, absence from the ten most prevalent sick leave malignant diagnoses generated more than half of the all-malignant losses. Additionally, the indirect costs of breast cancer absence solely was as much as 15% of losses from all the diseases analysed here.

The detailed estimates for all individual ICD-10 codes and for each year of the period analysed are presented in Supplementary Table 4 and Supplementary Table 5.

**Figure 2:**
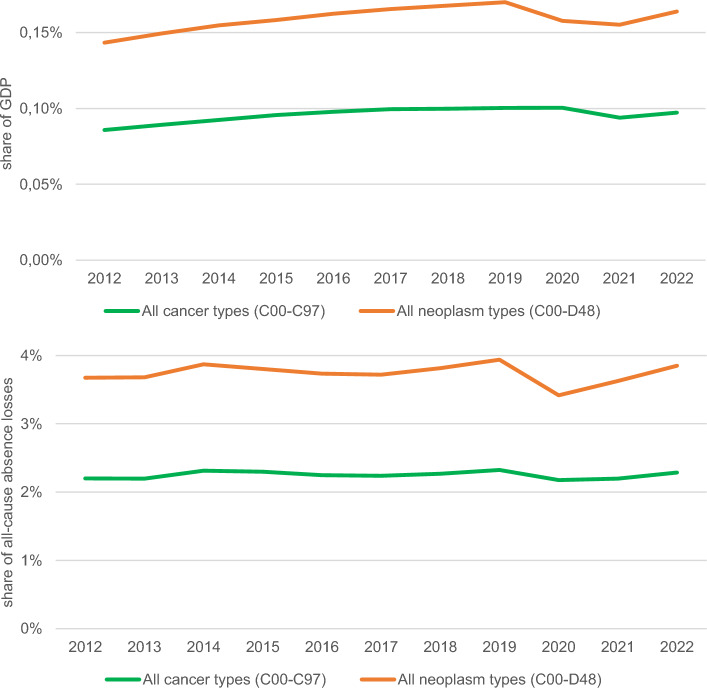
Time trends in relative measures of productivity losses associated with neoplasm-related work absence in Poland 2012–2022.

Sick leave work absence related to all neoplasms combined generated productivity losses from 0.143% GDP in 2012 to 0.170% GDP in 2019; this share declined sharply in the COVID-19 pandemic period of 2020–2021 (0.155–0.158%) and increased subsequently to 0.164% in 2022. For malignant neoplasms solely, the respective share was more stable; it increased from 0.086% of GDP in 2012 to 0.100% throughout the 2017–2020 period and declined only in 2021, the second year of the pandemic (0.094%) (Fig. 2).

**Figure 3:**
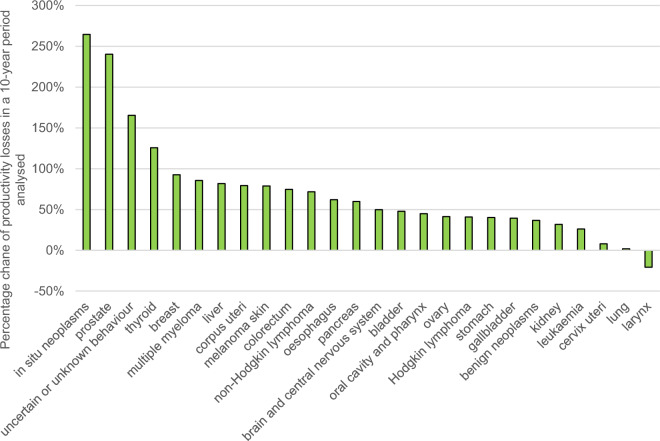
Percentage change of productivity losses associated with neoplasm-related work absence in Poland 2012–2022.

The time-trend analysis of the indirect costs shows that the productivity losses from in situ neoplasms and prostate cancer were more than triple in 2022 compared to 2012. For neoplasms of uncertain or unknown behaviour and thyroid cancer, the respective cost more than doubled throughout the period. Generally, for all but one (larynx cancer) analysed groups of malignant diseases the real losses in 2022 were higher than in 2012 (Fig. 3).

## Discussion

### General absence trends

This study analysed productivity losses (indirect costs) resulting from neoplasms-related sick leave absence in Poland in a decade-long period of 2012–2022. The analysis of social insurance data shows that, throughout the period, the number of absence days increased by 20.2% for all neoplasms (7.2 million to 8.7 million) and 19.2% for all cancer diagnoses (4.3 million to 5.2 million). These growths are higher than in all-cause absence (15.3%) and this shows the growing magnitude of neoplasms’ work absence burden relative to other diseases. For some commonly diagnosed malignant neoplasms like prostate, breast and colorectum cancer, these increases are even higher and they exceed 25%. The growing number of neoplasm-related work absenteeism may result from various factors. Epidemiological and demographic trends make neoplasms a more common disease^26–28^ and this is reflected in the growing absence in terms of episodes and workdays lost. Additionally, resultant of increasing labour shortages in Poland, there are incentives to continue employment after reaching retirement age and the effective labour market exit age in Poland keeps increasing (2010–2020 change: 59.6 to 62.2 years in males and 57.8 to 60.1 years in females^29^). Consequently, a higher number of senior people work and, because they more often develop cancer than the young, we observe an upward absence trend. This tendency can also result from other labour market factors like increasing employment in the economy (2012–2022 change: 15.6 to 16.7 million) or a dynamic decrease in unemployment rates (2012–2022 change: 13.4% to 5.2%). The latter factor might translate to higher absence rates because when unemployment is low, employees’ decision to take sick leave is less affected by the fear of job loss^30,31^ and absence rates rise.

Interestingly, in contrast to sick leave absence and the incidence of cancer in Poland (the latter not discussed here; for details see^26–28^), the time trend of neoplasm-related long-term inability to work exhibits a declining tendency. The number of disability pensions granted by the SII for all neoplasms (all cancers) patients was 30.3 thousand (28.1 thousand) in 2013 and it declined to 23.7 thousand (21.8 thousand) in 2022. Also, in four of five cancer diagnoses characterised by most disability pensions issued, a clear downward trend was apparent throughout the period (see Supplementary Fig. 1). Growing sick leave absence accompanied by decreasing disability rates might suggest that oncological treatment becomes more effective in limiting disability. Possibly, nowadays some of those cancer patients who would not be able to continue to work a decade ago, are now capable of ongoing employment owing to more effective treatment and their health deterioration results only in absence, not disability^32^.

### General productivity losses findings and comparison to previous studies

The findings show that short-term absence due to cancer and non-malignant neoplasms resulted in considerable losses for the economy and the resulting indirect costs (in real terms) increased by 66.1% in the period analysed for all neoplasms (2012–2022 change: €583 to €969 million). Again, the dynamics of this burden were higher than in all-cause absence (58.6%). The neoplasm-related absence losses were equivalent to 0.143% of GDP in 2012 and this share raised to 0.164% of GDP ten years later. Present results are in line with previous estimates from Poland; sick leave indirect costs from all neoplasms were €570 million in 2013^25^ (€593 million in this study) and €504 million in 2009^17^ (the year not analysed here). Some minor estimate differences arise from methodological choices, e.g. both previous studies used gross value added as a productivity measure while I used adjusted GDP as explained above.

This study used the classification of cancers by site as applied in the research on productivity losses from cancer mortality in Europe^11^. This approach intended to test whether the cancers that lead to high mortality losses are the ones that also account for the greatest work absence losses. Although the European study^11^ does not provide country-specific estimates by cancer site, I obtained data on Poland's losses for 23 cancer sites from the corresponding author (Dr Ortega-Ortega) of the article, who estimated and kindly agreed to share these for comparison. For this comparison, only paid productivity losses from the study^11^ were used because my estimates refer to formal economy losses and do not account for the burden due to informal activities referred to as ‘unpaid’ losses by Ortega-Ortega et al.^11^. There is a quite strong correlation (+ 0.494) between the total productivity losses from mortality and total productivity losses from sickness absence when analysing 23 cancer sites (data for 2018 was used in both loss categories). Therefore, a number of cancers that are ranked high in mortality losses also generate substantial absence losses, and other neoplasms are relatively low-burdening in terms of both absence losses and mortality losses. This is true for, e.g., breast cancer (ranking 3^rd^ in mortality and 1^st^ in absence losses), colorectum cancer (2^nd^ in both mortality and absence), lung cancer (1^st^ and 3^rd^, respectively) and multiple myeloma (20^th^ and 19^th^) or Hodgkin Lymphoma (21^st^ and 20^th^). On the other hand, some cancers that generate large absence losses (prostate—ranked 4^th^, thyroid—5^th^, bladder—7^th^) were ranked much lower in the mortality costs hierarchy (ranks 19, 23, 17, respectively). Considering the per death / per absence episode comparison of Poland’s productivity losses generated by cancer, there is a lower and negative correlation between the two categories (-0.234). Therefore, cancers generating high absence losses like lung, oesophagus, and larynx (ranking 2^nd^ to 4^th^, respectively) are ranked low in mortality losses (21^st^, 10^th^, and 14^th^, respectively). On the other hand, Hodgkin lymphoma, which is by far the most burdening in terms of mortality losses ranks 20^th^ in the absence losses and similar patterns apply to non-Hodgkin lymphoma, leukaemia, and melanoma skin (see Supplementary Table 6). Altogether, the comparison of absence losses with mortality losses by cancer sites in Poland shows that some cancers generate relatively large losses in one of the loss categories and modest economic burden in the other. Therefore, drawing policy conclusions on losses from a single indirect cost category (e.g. absenteeism) might be insufficient because the magnitude of losses resulting from a particular cancer might be different once other cost categories (e.g. mortality) are accounted for.

To give a broader perspective, all-neoplasm productivity losses from sick leave absence are lower than the same cost category in cardiovascular diseases in Poland which generated an average burden of €911 million in 2015–2017^21^ (here: €741 million, on average, for the respective years). Importantly, the burden of short-term work absence associated with neoplasms is relatively low as compared to other groups of conditions. This study analysed neoplasms solely; however, the previous study using 2013 data from Poland^25^ shows that diseases of the respiratory system or musculoskeletal system (mental disorders) generated indirect costs of absence four (two) times higher than neoplasms. This clearly shows the differences between neoplasms and the three above-mentioned groups of diseases in terms of their economic burden. Particularly, cancer generates relatively modest productivity losses in terms of sick leave absence, it rather leads to higher losses resulting from premature mortality and long-term inability to work as shown in a study of breast, cervix uteri and ovary cancer in Poland^33^. In contrast, mental disorders or musculoskeletal diseases are characterized by low mortality but higher rates of morbidity leading to greater work absence as compared to neoplasms. Considering spending on cancer treatment in Poland, the public expenditure on neoplasms was around €2.8 billion in 2022^34^ and these direct costs were almost triple the productivity losses resulting from short-term absence estimated here.

### Neoplasm site-specific absence and productivity losses

An important contribution of this study is the analysis of losses from site-specific cancers and single neoplasm diagnoses. A cancer site generating the heaviest economic toll was breast; breast cancer resulted in indirect costs of €146 million in 2022 and it was almost double the respective cost 10 years earlier. The magnitude and rising trend of this cancer cost results from the fact that breast cancer is the most common malignant neoplasm in Poland and its incidence is on a steadily rising trend. Moreover, a recent analysis shows that the average annual increase in this cancer incidence in Poland is the highest in the group of ten countries of the region^27^. This unfavourable tendency might be associated with low breast cancer screening; the 2019 figures show that 26.1% of women aged 45–64 in Poland had never undertaken breast examinations by X-ray. Importantly, this share increased by 3.6 percentage points from the time of the previous survey (2014) and was 8.8 percentage points higher than the European Union average value^35^.

The other important cancers in terms of absence losses were malignant neoplasms of colorectum (€63 million in 2022; 74.5% increase in 2012–2022 period), lung (€36 million; 1.7% increase), prostate (€50 million; 240% increase), and thyroid (€22 million; 126% increase). For colorectal cancer, again, we observe low adherence to preventive programmes; as much as 80% of the Polish population aged 50–74 in 2019 had never participated in colorectal cancer screening, whilst the respective share for neighbouring countries (39.7% in Slovakia and 33.6% in Czechia) or the EU average (48.7%) was notably lower^36^. Considering lung cancer, there was hardly any increase in the real indirect costs of sickness absence and this relatively favourable situation arises from the incidence trend in this disease. Particularly, resulting from declining tobacco consumption among men in Poland, we observe a dynamically diminishing incidence of lung cancer, and this trend offsets the increasing incidence in women^27^. Prostate cancer absence losses are characterised by the greatest dynamics among all cancer sites in the period analysed and this reflects adverse epidemiological trends of incidence; Poland had the largest increase of this cancer cases across 10 analysed countries in the 1990–2019 period^27^.

This study analysed not only the indirect costs of malignant neoplasms but also of benign neoplasms. The results show that the cost of short-term absence from benign neoplasms constitutes a large proportion of all-neoplasm losses. Particularly, the absence from leiomyoma of uterus (D25) resulted in productivity losses of €103.5 million in 2022, almost nine times higher than respective losses due to cervix uteri (C53). Similarly, benign neoplasm of ovary (D27) led to an economic cost of €40.0 million in 2022 which was almost double the burden of ovarian cancer (C56). This shows that for ovarian and uterus neoplasms, economic losses associated with sickness absence are concentrated in non-malignant cases. Therefore, to provide a broad picture of neoplasm-related economic losses, non-malignant diagnoses should be accounted for.

Interestingly, the estimates of per absence losses lead to different conclusions than the total losses analysis. The absence episode of brain and central nervous system cancer led to losses of €3,202 (2022) and no other neoplasm resulted in a comparable indirect cost. The other most burdening diagnoses in terms of average episode loss were neoplasms of lung, oesophagus, larynx, prostate, and oral cavity and pharynx, all leading to the cost of €2,625 to €2,731 per episode. These findings suggest that the less prevalent cancers (brain and central nervous system, oesophagus or larynx) which might be under-prioritised due to their lower epidemiological magnitude, are in fact the ones that lead to large economic losses per case^11^. On the other hand, prostate cancer and lung cancer are the ones that are costly in terms of both total and average losses, and this suggests that they should be prioritised in public health actions aimed at limiting health and economic burden. Unsurprisingly, per episode losses were higher for all cancers than all neoplasms and all causes combined.

### COVID-19 pandemic, sickness absence and productivity losses

The COVID-19 pandemic resulted in serious disruptions in health systems' capabilities to provide timely and effective health services^37^. The pandemic restrictions were associated with a widespread detrimental impact on cancer care in terms of delayed diagnoses, reduced service delivery, restricted access to medications, and missed cycles of therapies among others^38,39^. Consequently, around 20% decrease in the number of cancer diagnoses was observed in Poland in 2020, and this drop was even deeper in the April–May period when the most restrictive anti-SARS-CoV-2 policies were in place (e.g. 26% decline in colorectal cancer diagnoses)^40^. This under-diagnosis of neoplasms has significantly changed the trend of disease detection and delayed effects of these disturbances will likely be observed in the coming years^41^.

Accounting for the above trends, one would expect declining rates of neoplasm-related absence in the pandemic years. Interestingly, in the first pandemic year (2020) hardly any change in the number of absence days was observed for all malignant neoplasms combined (+ 0.3% compared to 2019) but a substantial decline in all neoplasms (-7.0%). For benign neoplasms, the respective change was as much as -22.7%. The above figures suggest that physicians were able to sustain the capability to issue sickness absence certificates during the pandemic inception but only for malignant neoplasm patients. Possibly, the health system was focused on more severe malignant neoplasms and there was less room for delivering the same service scope for benign neoplasm patients. Interestingly, there was a considerable increase in the number of absence days resulting from hematologic cancers, i.e. lymphoma (both Hodgkin and non-Hodgkin) and leukaemia in 2020. Some evidence suggests that SARS-CoV-2 might induce hematologic malignancies and their remission^42,43^; however, after a large increase of absence cases in 2020 in these diseases, we observe a decreasing trend in the two following years of the pandemic. On the other hand, the deepest drop in absence cases in 2020 among malignant neoplasms was observed in lung cancer (-11.6%) and it was followed by two years of continuous decline. This tendency reflects a general time trend of diminishing incidence of lung cancer among men.

The impact of the pandemic on cancer-related losses associated with sickness absence is reflected in a 7.4% decline in indirect costs of all neoplasms in 2020, but no changes were identified in the respective category of all malignant neoplasms combined. Clearly, the patterns of productivity losses reflect the tendencies observed in absence days; however, they are additionally strengthened by economic fluctuations, particularly the real GDP decline in 2020.

### Study limitations

The following limitations of the analysis should be acknowledged. Firstly, the study only accounts for one category of neoplasm-related productivity losses, namely, short-term work absence. With this approach, only a fragmented picture of the indirect costs of disease arises as no categories of inability to work, mortality or presenteeism were under scrutiny here. However, the above categories, particularly mortality losses, have been investigated in previous studies and my aim here was to provide a detailed (all neoplasm diagnoses as classified in ICD-10), dynamic (a decade-long) investigation with a thorough focus on work absence solely. Secondly, the choice of the human capital method as a basis for losses estimation is sometimes criticized in favour of the friction cost method (FCM)^18,19^. Yet, this study only accounts for short-term absenteeism; therefore, the HCM seems to be a more reasonable choice than FCM because a replacement of an employee who is absent only temporarily is unlikely^22^. Thirdly, only absence cases registered in the social insurance system were accounted for here and, because unrecorded episodes (e.g. using holiday instead of formal absence) are not included, this fact underestimates the real burden. The magnitude of this underestimation is difficult to assess. Fourthly, the analysis used average values of labour and economic model inputs and this might bias the results; e.g. neoplasm patients’ wages might deviate from average market values. Unfortunately, the direction and magnitude of this deviation are not evaluable. On the other hand, the use of average values provides a more generalized and representative understanding of the phenomenon and can help smooth out the impact of outliers and variations of individual data. Finally, the research only accounts for productivity losses in the formal sector as no data on unpaid costs borne due to cancer were evaluated here. This is because no data is obtainable on these diseases’ impact on daily informal activities. Unpaid productivity losses can be evaluated with more certainty for mortality losses as it is clear that a prematurely deceased person will not perform any activities^11^; yet, such an approach is not appropriate for short-term absence losses.

## Conclusion

This study analysed decade-long trends in incidence and productivity losses associated with all neoplasm diagnoses in Poland. It found that both absence and related economic losses increased steadily over time. Consequently, investing in effective public health policies that tackle neoplasms have the potential to reduce not only the health burden but also economic losses. This is particularly important due to the predicted population ageing and the resulting decline of labour supply in the country.

### Supplementary Information

Supplementary Information 1.

Supplementary Information 2.

## Data Availability

The data used for this analysis is publicly available with no restrictions from the websites of the Social Insurance Institutions statistical portal (https://psz.zus.pl/en/) and Statistics Poland (https://stat.gov.pl/en/). Moreover, the detailed data on absence days is shown in the Supplementary tables file.
