# Supplementary material for: Productivity losses from short-term work absence due to neoplasms in Poland
Source: Sci Rep. 2024 Feb 8;14:3289. doi: 10.1038/s41598-024-53878-4 (PMC10853257; doi:10.1038/s41598-024-53878-4)
Supplement: Supplementary file 1 — Supplementary Information 1. [file 41598_2024_53878_MOESM1_ESM.pdf]

Title: Productivity losses from short-term work absence due to neoplasms in Poland

Supplementary Fig 1. Number of disability pensions granted by the Social Insurance Institutions to cancer patients in Poland 2013-2022

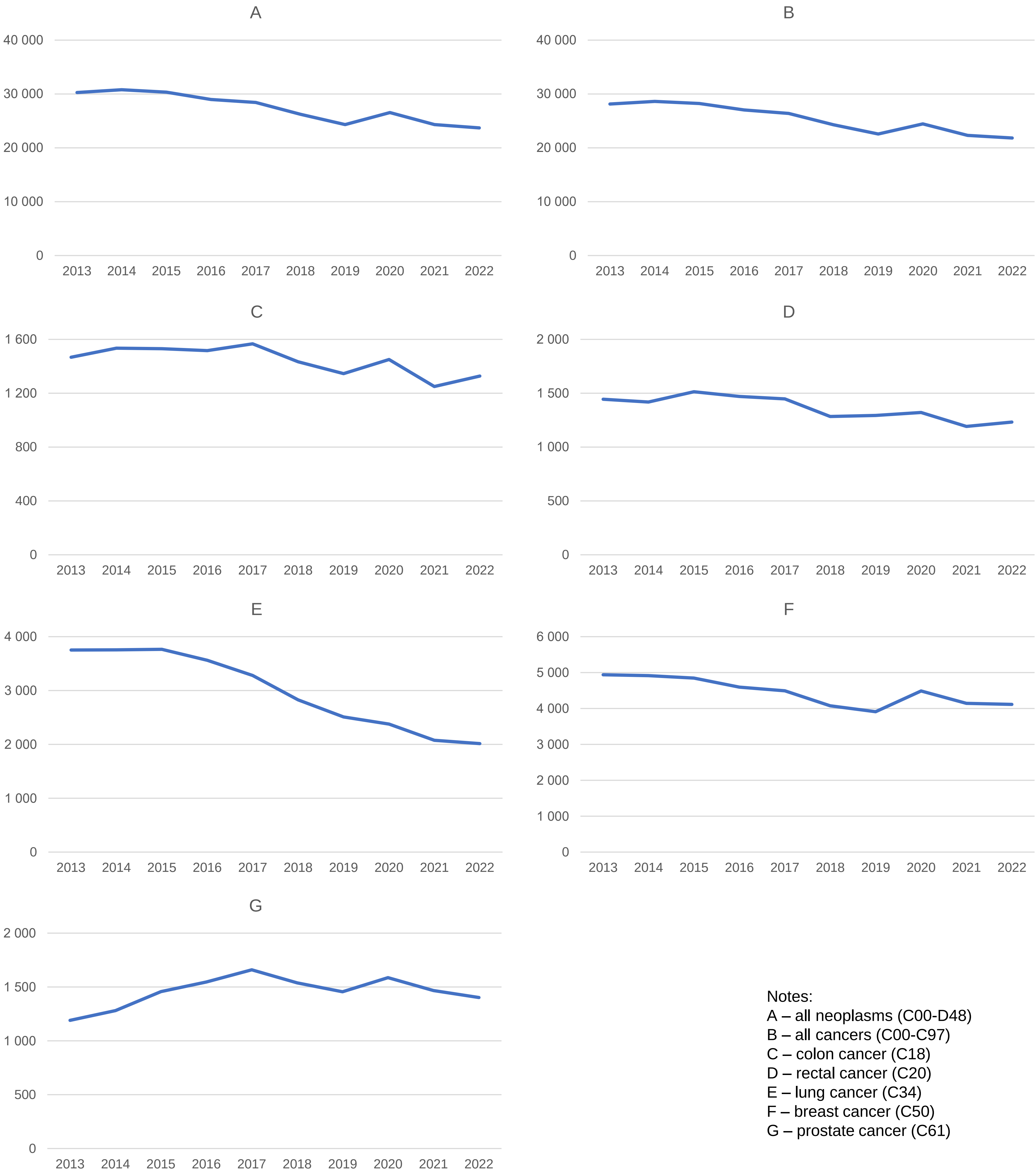

Notes:
A – all neoplasms (C00-D48)
B – all cancers (C00-C97)
C – colon cancer (C18)
D – rectal cancer (C20)
E – lung cancer (C34)
F – breast cancer (C50)
G – prostate cancer (C61)
