# Supplementary material for: Productivity losses from short-term work absence due to neoplasms in Poland
Source: Sci Rep. 2024 Feb 8;14:3289. doi: 10.1038/s41598-024-53878-4 (PMC10853257; doi:10.1038/s41598-024-53878-4)
Supplement: Supplementary file 2 — Supplementary Information 2. [file 41598_2024_53878_MOESM2_ESM.pdf]

Title: Productivity losses from short-term work absence due to neoplasms in Poland

Supplementary table 1. Neoplasm-related number of work absence days by single ICD-10 diagnoses in Poland in the years 2012-2022

	Number of absence days										
	2012	2013	2014	2015	2016	2017	2018	2019	2020	2021	2022
C00	2,687	2,445	2,673	3,691	3,462	2,549	2,947	2,687	2,274	3,158	3,201
C01	8,639	9,267	8,782	8,169	9,191	7,766	7,877	7,392	7,320	7,237	8,439
C02	13,559	15,756	16,770	17,036	19,205	22,105	21,891	19,975	20,983	20,366	20,616
C03	4,843	4,543	5,023	5,406	3,962	4,342	3,168	3,807	3,617	3,605	3,395
C04	13,639	13,798	15,234	17,703	21,265	19,454	18,583	17,535	16,588	17,443	19,106
C05	5,754	6,038	5,331	5,594	6,845	6,595	6,309	4,110	4,981	5,938	4,236
C06	8,195	7,055	7,444	8,194	8,240	9,750	13,274	12,177	8,683	9,638	10,621
C07	9,305	9,508	7,327	7,838	8,657	8,056	9,091	10,062	11,311	10,482	9,872
C08	3,997	4,036	3,770	4,118	4,127	2,733	3,875	4,093	2,970	3,885	2,336
C09	21,383	19,227	23,226	24,958	20,600	28,387	28,666	26,419	23,552	25,657	26,248
C10	11,735	9,978	8,747	10,069	10,303	9,920	11,444	8,152	7,862	8,606	8,239
C11	11,724	8,892	9,010	10,498	11,430	11,553	10,717	8,721	8,406	11,221	7,437
C12	2,980	3,012	4,094	4,399	4,980	6,054	4,990	4,570	4,133	3,358	3,888
C13	11,871	13,468	11,646	11,221	10,837	11,172	11,516	9,250	9,061	6,361	8,796
C14	2,898	3,395	2,578	4,274	3,789	3,874	3,900	2,552	2,751	2,841	3,267
C15	35,146	33,559	34,928	37,865	42,654	43,560	48,126	40,475	39,518	35,089	41,179
C16	136,445	131,840	138,500	145,901	149,392	145,211	142,478	142,893	133,074	131,999	138,440
C17	8,758	10,252	9,629	9,722	11,544	12,661	13,356	11,011	11,507	11,291	11,984
C18	232,090	254,191	251,679	285,955	292,898	302,829	292,875	306,541	303,423	299,567	302,045
C19	41,158	45,584	50,197	51,428	55,560	53,036	57,181	55,352	55,847	54,520	57,810
C20	165,177	171,808	172,685	181,886	194,849	196,919	190,526	199,315	181,471	190,855	192,708
C21	8,530	11,465	10,554	11,842	9,188	11,963	11,858	11,676	10,281	12,486	12,092
C22	25,206	25,613	26,647	25,172	29,188	30,723	31,075	27,923	27,375	28,821	33,165
C23	12,826	11,520	14,395	12,911	14,690	11,589	11,254	10,095	10,784	8,714	9,577
C24	9,432	8,948	9,706	11,870	13,852	13,753	12,921	13,526	14,555	12,719	12,888
C25	72,732	66,455	80,652	81,339	84,049	85,303	90,309	87,989	89,713	85,737	84,117
C26	7,403	7,390	6,927	7,209	8,303	6,982	4,446	2,617	3,246	2,873	2,040
C30	4,016	4,667	4,361	5,060	5,074	4,460	4,737	3,604	4,077	4,526	4,629
C31	6,259	7,362	7,276	5,957	5,024	7,374	6,395	5,994	7,140	5,376	5,927
C32	77,697	68,743	75,591	70,562	71,491	69,560	68,386	59,415	57,777	58,108	44,543
C33	845	1,671	1,201	1,332	1,252	1,054	616	728	549	524	774
C34	439,179	438,578	448,075	464,696	448,280	426,030	413,124	383,290	338,977	318,830	323,216
C37	2,401	1,820	1,895	2,907	4,277	2,957	3,617	3,882	3,993	4,489	3,781
C38	5,312	6,301	7,329	6,053	6,713	6,967	5,294	6,187	5,019	4,099	5,975
C39	1,826	2,520	2,706	1,957	2,875	1,933	2,399	1,388	1,522	1,110	1,343
C40	13,012	13,559	12,774	16,566	15,899	17,511	13,681	13,608	10,627	11,457	11,245

C41	10,390	9,178	7,948	11,794	12,666	12,094	13,294	12,511	11,631	11,005	13,429
C43	77,413	80,851	82,410	90,485	91,613	101,548	92,830	98,630	105,059	100,910	100,181
C44	40,367	36,034	41,080	41,699	37,450	41,126	45,161	48,631	40,393	42,381	49,161
C45	6,036	6,356	6,610	5,823	5,341	5,474	5,853	5,046	6,649	6,712	7,097
C46	511	692	1,053	654	534	1,119	842	829	1,290	601	882
C47	928	986	1,208	969	794	1,172	1,074	540	884	758	984
C48	8,698	13,030	8,394	9,815	11,848	11,958	10,837	12,587	12,270	11,821	10,698
C49	38,097	38,253	40,591	43,993	44,733	43,465	45,295	51,048	49,742	49,234	48,560
C50	942,255	974,497	1,021,195	1,074,620	1,099,652	1,129,149	1,173,367	1,268,092	1,317,535	1,247,172	1,313,504
C51	8,584	7,085	8,995	8,711	8,278	7,721	8,813	7,527	9,446	6,940	8,427
C52	2,876	2,503	3,088	2,609	2,737	2,473	2,870	2,774	2,538	1,898	3,155
C53	138,187	127,013	131,832	126,441	123,782	124,693	114,071	107,326	103,274	106,808	107,996
C54	123,902	134,387	141,568	149,693	157,070	155,102	158,268	165,140	156,996	154,677	160,723
C55	2,823	2,695	2,468	2,415	2,810	2,269	2,015	1,322	1,727	1,986	1,428
C56	185,182	188,824	195,274	189,980	201,673	197,921	203,791	214,068	209,090	191,814	189,449
C57	4,483	5,023	4,844	5,338	4,949	4,126	5,276	4,860	4,944	4,716	5,176
C58	1,485	2,099	1,490	1,871	2,191	1,309	972	1,976	1,847	1,343	1,154
C60	7,220	7,956	9,893	8,449	9,444	9,593	11,543	9,067	9,736	9,348	10,012
C61	182,757	214,059	248,457	297,883	334,684	375,534	388,143	408,950	432,101	379,214	449,985
C62	85,649	87,991	84,404	86,695	83,788	92,880	94,002	92,491	90,444	83,209	79,844
C63	1,704	1,696	2,019	1,198	1,284	1,510	908	1,298	908	719	1,046
C64	141,905	155,167	146,268	159,934	155,604	152,352	159,184	156,099	143,955	141,968	134,582
C65	7,169	6,789	7,251	7,378	7,544	7,767	7,634	6,728	5,816	6,284	7,518
C66	3,246	3,046	2,949	3,641	3,193	3,768	3,920	3,527	3,904	2,630	3,188
C67	143,353	146,135	149,140	158,174	163,738	169,287	174,345	167,463	171,455	157,208	153,284
C68	1,524	1,634	1,596	626	1,325	1,097	1,016	1,057	1,243	822	652
C69	15,466	16,080	18,558	17,195	19,017	20,488	19,336	21,452	19,586	17,012	20,663
C70	3,797	3,969	3,436	4,851	4,476	5,095	3,558	3,695	1,751	2,401	3,235
C71	133,622	136,013	144,473	155,225	147,901	158,912	148,819	140,146	148,483	142,086	147,246
C72	7,674	8,512	7,899	8,002	6,603	6,945	8,324	6,863	5,403	5,327	6,720
C73	123,683	132,248	142,442	178,246	198,315	200,221	204,918	215,748	206,223	198,307	201,954
C74	3,934	4,276	4,307	4,959	2,991	5,247	5,013	5,402	3,906	2,451	3,668
C75	3,217	3,732	2,810	3,437	5,813	5,224	4,427	5,892	9,157	6,229	9,820
C76	10,315	9,890	9,923	11,848	8,585	9,880	9,082	9,293	9,729	8,652	8,285
C77	8,029	8,628	9,008	8,453	8,731	11,982	11,982	10,621	10,725	12,244	10,452
C78	21,553	21,119	24,376	27,751	32,554	32,362	33,893	28,852	29,823	28,735	28,580
C79	24,234	23,738	25,427	25,013	29,315	30,896	30,776	31,780	30,101	30,010	29,860
C80	29,967	30,610	33,748	29,823	26,021	24,104	27,041	23,284	25,554	22,606	19,141
C81	61,489	60,153	63,012	64,073	67,065	64,723	64,667	61,324	67,653	64,874	62,675
C82	20,677	22,049	25,108	22,482	23,257	22,712	26,068	23,933	32,773	29,944	30,362
C83	45,770	47,689	47,425	45,439	48,150	47,341	38,595	32,979	36,215	34,745	40,356
C84	10,687	10,835	10,417	11,739	10,622	13,115	9,403	7,941	11,003	8,541	8,851
C85	32,283	39,639	45,189	51,652	54,577	61,579	64,058	63,740	73,161	60,580	60,511

C88	2,293	1,894	2,074	2,523	2,954	3,021	3,323	4,180	2,652	3,495	3,674
C90	40,294	42,073	52,795	53,458	55,669	60,271	62,310	55,137	58,348	54,955	53,532
C91	65,814	62,864	71,870	73,481	78,821	73,556	75,244	70,486	85,971	64,879	63,088
C92	48,363	55,384	59,107	53,701	50,566	59,271	59,368	38,329	47,174	38,396	42,667
C93	1,825	1,679	1,866	1,973	2,078	2,322	1,561	919	1,449	1,019	1,470
C94	2,737	3,102	2,812	2,931	3,364	2,410	1,934	1,502	1,666	1,976	1,054
C95	550	1,048	1,332	1,191	637	629	452	413	423	515	568
C96	5,156	4,729	5,740	7,415	4,994	5,420	4,124	2,807	3,669	2,083	2,290
C97	1,756	2,189	1,775	1,395	1,782	1,840	1,306	684	646	388	583
D00	1,106	1,052	1,305	1,376	1,650	1,788	1,080	1,197	520	632	1,222
D01	n.a.	n.a.	n.a.	n.a.	n.a.	n.a.	n.a.	n.a.	253	2,083	2,444
D02	1,259	1,134	1,414	1,424	1,447	1,393	1,241	589	488	721	1,081
D03	1,583	1,186	1,624	1,645	1,318	1,655	1,924	2,024	1,653	1,433	1,535
D04	2,072	2,120	1,917	1,820	2,300	2,148	2,046	1,710	1,121	1,353	1,413
D05	9,856	12,920	13,645	17,341	20,431	20,287	23,564	30,404	26,234	30,312	37,848
D06	4,768	5,055	4,497	4,160	5,646	4,988	6,436	8,488	8,208	8,466	8,844
D07	929	981	1,204	1,571	1,271	2,842	4,448	4,425	4,349	4,143	3,968
D09	3,133	2,607	3,157	2,900	3,802	3,685	4,473	5,766	6,900	7,534	6,818
D10	9,334	9,845	11,281	10,355	10,119	11,744	9,295	9,629	7,359	7,203	8,962
D11	17,765	21,586	21,457	26,705	27,595	34,766	32,429	32,760	29,634	27,658	33,388
D12	33,468	37,790	39,767	41,527	39,654	41,028	42,398	40,539	30,588	28,635	31,794
D13	19,766	21,403	21,294	22,868	20,803	20,846	20,515	16,374	13,623	12,399	17,161
D14	14,141	13,504	14,003	16,235	14,158	18,100	15,045	14,815	11,700	12,259	14,086
D15	10,291	8,193	7,182	9,175	6,794	8,650	7,848	7,307	5,825	7,268	7,149
D16	71,697	77,118	80,634	84,874	86,844	88,204	92,379	90,431	68,024	70,070	78,039
D17	55,335	57,450	62,160	66,244	74,623	73,395	75,121	77,775	48,630	61,924	82,652
D18	13,012	15,211	17,525	19,317	19,513	21,534	20,730	20,204	15,730	17,549	16,634
D19	411	560	661	568	561	798	392	344	379	194	198
D20	6,394	6,092	5,513	6,082	7,163	6,481	5,584	3,271	3,290	3,862	4,529
D21	117,274	125,731	127,777	130,260	146,120	149,405	148,876	155,500	106,736	125,194	148,699
D22	36,624	41,144	43,466	50,724	54,089	54,704	55,710	60,872	40,502	46,388	59,720
D23	133,074	133,726	144,359	146,213	151,190	149,836	147,817	149,048	99,673	111,880	125,915
D24	56,592	46,776	43,901	39,126	40,455	37,429	33,358	34,015	25,851	26,014	26,436
D25	962,642	965,391	962,124	933,557	909,758	892,589	929,477	955,230	726,710	825,877	928,752
D26	13,598	16,617	18,947	21,083	21,711	19,643	18,379	13,911	14,361	12,481	13,471
D27	424,988	430,642	436,574	441,886	448,846	437,038	440,813	436,537	356,330	362,181	358,940
D28	11,022	11,743	12,504	12,025	10,939	9,681	8,885	8,557	7,093	5,860	5,825
D29	4,353	3,354	2,977	3,447	3,420	3,407	3,043	2,177	2,233	1,714	2,004
D30	12,514	10,023	10,237	10,391	11,241	10,488	9,101	7,441	6,356	6,747	7,473
D31	4,336	4,947	3,479	2,846	3,881	2,903	3,179	4,331	4,361	4,815	3,598
D32	37,451	37,647	41,759	42,900	51,691	52,187	53,809	52,898	47,926	47,823	55,945
D33	80,487	80,269	81,496	83,892	86,255	86,715	91,156	83,979	81,363	73,059	79,070
D34	17,561	15,786	18,851	16,223	13,699	12,993	12,602	11,163	8,647	9,954	8,810

D35	36,877	43,238	40,420	43,816	44,314	48,799	50,003	50,767	43,985	51,200	54,565
D36	6,698	9,192	8,714	8,570	8,239	10,031	8,042	6,380	6,470	6,960	8,861
D37	130,022	140,778	157,279	178,909	194,472	225,768	245,500	261,573	248,918	260,415	274,721
D38	154,264	170,924	187,784	183,574	193,055	204,053	215,434	219,156	197,061	217,534	222,019
D39	11,796	11,960	14,021	16,342	18,389	23,168	35,376	38,957	41,890	50,148	53,395
D40	13,885	16,541	21,251	24,676	28,579	32,930	40,242	45,949	43,660	48,685	52,766
D41	48,728	56,950	70,079	89,832	104,265	115,311	125,131	141,132	124,799	136,546	146,500
D42	4,816	4,721	6,585	6,595	8,405	7,926	6,901	7,089	4,010	4,654	4,271
D43	68,574	82,303	84,987	89,373	87,802	90,066	102,729	96,775	90,939	100,224	97,858
D44	25,889	28,153	35,935	47,600	55,264	63,096	65,309	71,370	63,700	66,877	71,984
D45	5,975	5,790	7,646	8,674	9,117	9,094	8,497	8,832	12,437	7,399	9,615
D46	10,364	11,679	12,506	10,861	13,577	15,753	14,724	13,360	12,969	13,701	11,969
D47	18,271	19,843	24,749	25,814	29,318	35,313	39,229	40,764	47,867	45,308	42,947
D48	176,659	190,230	213,650	228,430	257,097	271,638	281,497	298,145	250,896	274,791	297,506

Notes: n.a. – not available. The names of diseases for each ICD-10 code are available in the World Health Organization website: https://icd.who.int/browse10/2019/en#/II.

Supplementary table 2. Neoplasm-related average length of work absence episode by single ICD-10 diagnoses in Poland in the years 2012-2022

	Average length of an absence episode (days)										
	2012	2013	2014	2015	2016	2017	2018	2019	2020	2021	2022
C00	21.7	19.4	22.3	25.5	23.2	20.9	21.7	22.0	20.5	19.0	21.5
C01	27.3	25.0	27.6	26.0	27.8	25.1	28.4	23.8	24.1	25.0	24.5
C02	26.2	24.2	24.5	24.3	25.5	26.2	24.5	23.0	22.1	24.1	22.8
C03	23.0	24.2	23.6	22.9	22.9	21.5	21.7	21.8	23.3	23.9	23.7
C04	27.3	28.4	27.1	26.9	28.1	28.3	29.5	25.1	25.2	24.7	24.9
C05	24.2	25.6	23.9	25.2	24.2	22.5	27.9	21.7	21.6	21.2	18.8
C06	23.2	25.9	24.6	24.4	26.9	26.4	28.2	24.6	23.5	24.8	24.4
C07	25.1	23.7	21.3	21.7	22.5	21.7	22.2	20.0	21.9	21.3	19.0
C08	20.7	17.9	19.9	19.4	17.9	17.4	19.7	21.9	18.1	22.9	20.7
C09	30.7	28.9	28.3	28.3	27.0	27.7	27.7	25.6	25.7	25.7	25.1
C10	28.9	26.2	27.3	25.8	26.6	26.6	26.3	24.2	23.6	25.4	23.8
C11	27.6	24.6	27.9	24.2	25.2	23.7	24.2	23.1	24.6	25.1	24.4
C12	31.7	27.6	28.2	26.5	30.9	29.4	27.3	26.6	25.8	25.2	24.3
C13	26.7	25.6	24.8	25.8	25.2	26.2	28.1	24.5	27.0	24.1	25.6
C14	20.3	21.0	24.3	24.4	20.9	23.1	23.2	23.2	21.3	24.7	25.9
C15	25.2	27.1	26.3	26.9	27.7	26.9	26.6	24.5	24.4	24.3	24.2
C16	26.9	26.5	26.8	26.6	26.3	25.7	25.2	24.2	24.5	23.6	21.8
C17	22.7	23.4	22.0	23.0	23.0	21.1	21.4	19.9	21.6	19.7	17.6
C18	24.2	24.4	24.0	23.7	23.5	23.7	23.7	22.6	22.9	21.7	20.8
C19	24.8	24.4	25.2	24.0	24.9	24.0	24.0	23.1	23.4	21.6	21.5
C20	26.1	26.0	25.3	25.1	25.1	25.3	25.1	24.1	23.6	23.1	22.7
C21	24.2	24.3	24.8	24.1	26.0	25.6	25.3	23.9	23.3	25.1	25.6
C22	22.9	22.1	23.6	23.7	24.9	23.9	24.2	22.7	22.8	22.9	21.7
C23	23.8	22.4	23.7	21.9	23.3	22.4	24.4	22.6	25.3	23.3	21.3
C24	26.8	25.3	24.2	24.2	26.3	24.0	25.6	24.5	25.3	21.5	21.8
C25	25.7	25.0	26.9	24.6	25.1	25.1	24.3	22.4	23.5	22.9	21.9
C26	22.8	22.7	25.4	24.0	23.6	23.0	22.2	23.4	22.9	21.8	24.9
C30	16.0	18.0	15.3	17.9	17.2	17.2	19.8	20.5	20.3	21.8	21.8
C31	29.2	26.4	27.6	26.2	24.3	27.4	24.5	23.5	23.0	23.9	24.4
C32	28.3	27.6	27.5	26.9	27.2	26.3	26.2	24.2	25.1	24.2	23.8
C33	22.2	24.6	23.1	26.6	27.8	27.7	23.7	19.7	23.9	12.5	16.8
C34	26.1	26.2	26.5	26.5	26.5	26.8	26.9	25.7	25.8	25.6	24.5
C37	23.3	24.9	19.9	25.5	25.0	19.6	23.8	26.6	24.2	22.9	23.3
C38	22.4	23.6	23.6	23.7	23.7	26.0	27.3	25.9	24.5	24.1	25.8
C39	21.7	19.8	20.5	20.2	24.0	19.7	20.5	21.4	21.1	23.6	22.8
C40	23.5	24.9	22.3	22.5	22.9	23.5	22.4	25.8	25.2	25.1	25.8
C41	25.8	28.5	24.8	27.9	27.1	28.6	29.0	25.0	25.8	23.3	24.8
C43	20.3	20.6	19.7	19.9	19.3	18.5	17.8	16.2	17.4	15.7	14.4

C44	15.8	15.4	15.8	15.3	14.1	14.7	14.8	14.2	14.3	13.1	12.7
C45	24.7	24.6	25.0	24.5	24.2	24.4	25.0	25.5	26.2	24.8	24.7
C46	23.2	21.6	22.9	18.7	21.4	16.0	14.5	14.1	15.5	10.5	15.8
C47	18.9	17.0	29.5	19.4	17.3	17.0	17.6	22.5	20.6	21.1	27.3
C48	24.1	24.1	22.3	22.1	22.9	21.7	20.1	20.0	21.3	19.9	18.2
C49	23.8	23.8	23.3	23.1	22.4	22.4	21.5	20.7	21.6	20.9	19.3
C50	24.7	24.4	24.1	23.9	23.6	23.3	23.2	22.2	23.5	21.8	20.0
C51	25.1	23.9	22.3	24.6	23.6	23.0	24.2	22.3	23.6	21.7	21.6
C52	22.5	22.8	22.5	20.9	22.4	21.1	23.7	26.2	23.7	24.6	21.9
C53	26.7	26.4	27.1	25.7	25.4	24.9	25.0	23.9	24.5	24.1	22.9
C54	25.5	25.4	25.1	24.6	24.4	24.1	24.7	24.4	24.6	23.6	23.1
C55	17.6	20.1	18.6	17.4	16.3	16.6	18.3	17.4	19.9	21.1	20.7
C56	23.0	23.3	23.1	23.0	23.2	22.5	23.1	22.2	22.7	22.0	20.8
C57	20.8	23.1	22.4	21.9	23.1	24.1	21.6	21.7	22.9	22.2	22.5
C58	20.6	17.8	19.1	16.6	17.5	20.8	21.1	16.2	18.1	14.4	14.2
C60	23.4	22.4	24.6	21.4	22.4	23.5	24.1	23.1	21.3	22.1	21.5
C61	24.6	25.4	25.2	25.0	24.9	25.3	25.0	24.4	24.7	24.2	23.7
C62	21.5	20.6	20.1	21.0	20.9	21.2	21.1	19.7	20.3	19.5	18.5
C63	20.5	17.5	19.8	20.7	17.6	20.7	16.5	20.6	21.1	21.1	17.4
C64	25.1	25.1	24.4	25.1	25.2	24.4	25.1	23.9	24.3	23.8	22.4
C65	22.7	23.3	24.6	23.4	23.8	24.4	24.0	22.4	24.3	23.0	24.7
C66	23.0	22.9	23.6	20.2	22.5	22.2	24.3	21.5	24.4	21.0	22.0
C67	19.1	18.9	19.0	19.4	19.1	19.3	19.0	17.8	19.0	18.1	17.0
C68	18.8	21.2	19.0	17.9	18.7	19.6	19.9	20.3	23.0	23.5	18.1
C69	22.0	21.0	21.0	19.8	20.7	20.1	19.6	20.4	20.4	18.6	18.6
C70	22.2	24.5	22.3	23.8	22.8	24.1	25.1	24.6	20.6	23.3	26.5
C71	29.5	29.9	29.7	30.0	30.3	31.2	31.0	28.7	29.0	28.5	28.8
C72	24.9	24.3	23.1	24.1	23.6	24.0	26.7	24.2	24.8	26.1	27.5
C73	19.0	19.0	19.2	19.4	19.4	19.1	18.8	18.4	18.5	17.7	16.9
C74	20.4	19.3	19.2	20.7	16.1	20.3	19.8	18.1	19.6	17.5	16.3
C75	15.6	15.3	16.6	15.6	18.6	15.1	13.7	13.5	17.1	12.3	13.8
C76	23.7	23.7	24.4	24.1	24.2	22.9	22.2	19.6	22.5	20.7	19.0
C77	23.1	24.0	24.0	22.8	22.5	22.4	24.7	21.9	21.1	21.9	19.8
C78	27.1	26.2	26.4	27.3	27.6	25.9	26.6	24.6	24.3	23.2	22.8
C79	27.7	27.6	27.2	27.1	27.7	27.0	27.3	26.8	27.3	27.0	25.3
C80	23.7	24.4	25.1	24.9	24.6	25.7	25.5	24.3	24.0	24.0	23.8
C81	21.1	19.8	20.4	20.3	20.6	20.8	20.6	19.9	20.8	19.3	17.8
C82	20.1	19.3	20.0	19.9	19.3	17.8	19.8	19.0	19.9	19.3	16.4
C83	23.1	23.3	23.8	23.9	24.5	23.7	23.5	21.4	23.0	22.1	22.1
C84	21.5	22.4	22.0	21.8	20.6	21.2	20.7	17.8	20.3	19.3	18.3
C85	23.1	23.4	23.5	24.6	24.3	24.6	23.7	22.0	21.9	20.8	20.2
C88	17.5	18.9	18.4	22.5	20.1	21.3	21.6	21.4	17.7	19.9	15.5
C90	23.6	22.9	24.5	24.6	23.6	24.6	24.4	20.6	22.7	21.4	20.5

C91	22.5	22.2	22.7	22.6	22.4	21.9	21.9	20.1	20.4	20.0	17.8
C92	22.6	23.6	22.8	22.9	22.1	22.8	20.8	15.6	17.6	17.3	16.2
C93	26.1	24.7	23.6	21.9	20.2	22.5	24.8	21.9	25.4	24.3	21.9
C94	16.6	15.4	16.3	15.6	16.3	11.6	13.9	16.7	19.6	15.9	13.2
C95	13.1	16.6	17.1	18.9	16.3	15.3	12.2	12.1	13.6	16.6	17.2
C96	19.6	17.3	18.2	18.7	16.2	16.9	17.1	15.9	16.5	14.9	13.6
C97	18.1	19.4	19.1	19.9	19.8	20.4	19.5	19.0	20.8	18.5	21.6
D00	12.7	11.0	13.2	13.0	14.1	11.8	12.0	16.6	12.7	12.9	14.9
D01	n.a.	n.a.	n.a.	n.a.	n.a.	n.a.	n.a.	n.a.	21.1	16.3	13.7
D02	10.9	9.9	12.5	10.4	10.3	11.5	11.8	16.4	14.8	15.3	22.1
D03	14.3	12.9	12.7	11.6	12.2	11.5	13.8	13.9	12.7	12.8	10.8
D04	11.3	10.5	10.1	11.4	11.7	11.5	11.4	11.0	10.5	10.7	9.2
D05	21.4	25.0	21.7	21.7	21.5	21.0	22.1	21.7	22.8	21.6	20.3
D06	18.9	16.3	17.8	15.2	16.3	15.3	14.9	16.0	16.4	15.4	15.2
D07	14.7	17.5	18.0	19.6	18.2	24.1	22.9	21.8	18.9	21.5	20.7
D09	21.2	19.8	19.6	17.7	16.5	18.9	19.3	20.4	21.0	19.4	19.9
D10	10.8	10.8	11.1	10.3	10.4	10.5	9.5	9.1	10.6	9.0	9.2
D11	16.8	16.4	16.5	16.9	16.9	16.9	17.1	16.2	16.3	16.3	15.8
D12	11.2	11.4	11.2	10.8	10.4	10.0	9.5	8.5	8.9	8.6	8.4
D13	15.9	16.9	16.7	16.4	15.2	15.0	14.8	13.0	14.8	13.8	13.6
D14	18.4	18.7	17.9	19.3	17.6	18.4	17.2	18.0	17.8	17.5	16.6
D15	24.2	21.2	20.2	20.4	20.2	22.0	21.4	20.5	20.7	22.0	19.2
D16	22.0	22.9	23.3	22.9	23.0	22.9	23.1	22.4	22.2	21.5	21.4
D17	10.6	10.6	10.6	10.7	11.0	10.8	11.0	10.6	10.7	10.3	10.3
D18	16.9	17.0	16.5	16.7	16.3	15.4	15.3	15.0	15.5	15.2	13.7
D19	10.3	10.8	12.2	13.2	11.7	14.8	10.3	11.9	11.8	16.2	15.2
D20	17.1	18.5	17.1	17.0	18.0	18.0	18.9	18.8	19.5	20.9	20.8
D21	12.6	12.4	12.5	12.6	13.1	13.3	13.2	12.9	12.9	12.3	11.9
D22	7.1	6.9	6.9	6.9	6.9	6.9	6.6	6.2	6.5	5.9	5.5
D23	8.7	8.5	8.5	8.4	8.5	8.3	8.3	7.8	8.3	7.8	7.3
D24	11.2	10.9	10.4	10.0	9.6	9.2	8.6	8.3	9.2	8.4	7.8
D25	25.6	25.4	25.1	24.9	24.4	24.1	23.6	22.8	22.4	21.7	21.3
D26	19.0	18.8	18.7	18.0	18.7	19.4	18.1	14.9	15.9	14.1	14.9
D27	22.3	22.1	22.2	21.9	21.6	21.4	21.1	20.3	20.1	19.9	19.3
D28	15.4	16.2	15.8	15.4	15.5	15.4	14.8	16.0	18.1	15.8	14.3
D29	16.7	15.7	15.1	15.7	14.5	15.3	14.2	14.6	16.5	13.9	13.5
D30	16.3	14.7	14.8	16.0	15.9	14.3	14.8	13.7	15.0	14.1	14.2
D31	12.1	12.8	14.1	14.0	14.8	12.2	11.2	10.7	11.9	11.7	9.8
D32	26.3	26.4	25.5	26.1	26.0	25.6	24.5	23.7	23.4	24.5	24.6
D33	26.3	27.0	25.8	25.8	25.1	25.6	26.5	24.2	25.0	25.3	24.9
D34	23.3	24.6	23.7	22.6	21.4	20.5	19.2	19.3	19.8	18.5	17.3
D35	16.9	16.7	16.0	15.2	14.8	14.9	14.2	13.2	14.3	13.9	12.7
D36	15.9	17.5	16.0	16.0	16.0	17.3	15.5	15.2	17.1	15.0	15.1

D37	17.2	17.1	16.7	16.3	16.3	16.3	16.2	15.2	16.2	15.8	14.9
D38	19.6	19.9	20.0	19.4	19.4	19.1	18.8	18.0	18.2	17.7	17.2
D39	15.1	14.9	14.8	14.6	14.9	14.6	16.4	15.8	16.6	16.6	16.6
D40	16.6	17.0	16.8	15.8	15.0	15.2	15.7	14.5	14.8	14.4	13.6
D41	18.5	18.5	18.5	18.9	19.1	19.0	18.7	18.1	18.6	18.5	18.2
D42	22.4	22.1	24.2	23.7	24.9	23.4	21.0	22.8	20.0	21.3	20.5
D43	26.0	26.6	26.5	25.7	26.4	26.0	26.0	24.9	24.9	26.0	24.7
D44	20.1	20.3	20.4	21.2	21.5	20.1	19.6	19.0	19.1	18.5	17.8
D45	11.4	11.7	11.3	11.7	11.4	10.3	10.6	9.9	12.7	9.3	9.4
D46	21.3	21.1	19.3	19.4	18.7	20.2	18.9	16.8	19.0	20.4	18.3
D47	15.5	15.6	16.0	15.3	14.9	15.4	14.3	13.0	14.6	13.7	12.2
D48	13.8	13.7	13.5	13.2	13.4	13.2	12.6	11.8	12.7	11.8	10.7

Notes: n.a. – not available. The names of diseases for each ICD-10 code are available in the World Health Organization website: https://icd.who.int/browse10/2019/en#/II.

Supplementary table 3. Year-to-year percentage changes in the number of absence days by cancer site in Poland in the COVID-19 pandemic period

	Percentage change		
	change from 2020 to 2019	change from 2021 to 2020	change from 2022 to 2020
oral cavity and pharynx (C00-C14)	-5.0%	3.9%	3.9%
oesophagus (C15)	-2.4%	-11.2%	4.2%
stomach (C16)	-6.9%	-0.8%	4.0%
colorectum (C18-C21)	-3.8%	1.2%	2.5%
liver (C22)	-2.0%	5.3%	21.2%
gallbladder (C23-C24)	7.3%	-15.4%	-11.3%
pancreas (C25)	2.0%	-4.4%	-6.2%
larynx (C32)	-2.8%	0.6%	-22.9%
lung (C33-C34)	-11.6%	-5.9%	-4.6%
melanoma skin (C43)	6.5%	-3.9%	-4.6%
breast (C50)	3.9%	-5.3%	-0.3%
cervix uteri (C53)	-3.8%	3.4%	4.6%
corpus uteri (C54)	-4.9%	-1.5%	2.4%
ovary (C56)	-2.3%	-8.3%	-9.4%
prostate (C61)	5.7%	-12.2%	4.1%
kidney (C64-65)	-8.0%	-1.0%	-5.1%
bladder (C67)	2.4%	-8.3%	-10.6%
brain and central nervous system (C70-72)	3.3%	-3.7%	1.0%
thyroid (C73)	-4.4%	-3.8%	-2.1%
Hodgkin lymphoma (C81)	10.3%	-4.1%	-7.4%
non-Hodgkin lymphoma (C82-C85, C96)	19.3%	-13.3%	-9.2%
multiple myeloma (C88, C90)	2.8%	-4.2%	-6.2%
leukaemia (C91-C95)	22.4%	-21.9%	-20.4%
in situ neoplasms (D00-D09)	-8.9%	14.0%	31.1%
benign neoplasms (D10-D36)	-22.7%	8.5%	20.4%
neoplasms of uncertain or unknown behaviour (D37-D48)	-8.4%	7.6%	12.9%
all cancers (C00-C97)	0.3%	-5.2%	-1.9%
all neoplasms (C00-D48)	-7.0%	-0.3%	5.2%
all diseases (A00-Z99)	7.2%	-6.3%	-6.9%

Supplementary table 4. Neoplasm-related work absence total productivity losses by single ICD-10 diagnoses in Poland in the years 2012-2022

	Total losses (thousand €)										
	2012	2013	2014	2015	2016	2017	2018	2019	2020	2021	2022
C00	216,764	193,929	217,273	309,911	300,812	237,298	287,161	277,874	234,331	343,985	356,735
C01	696,918	735,028	713,839	685,902	798,604	722,972	767,549	764,437	754,309	788,289	940,484
C02	1,093,821	1,249,713	1,363,139	1,430,411	1,668,718	2,057,854	2,133,099	2,065,697	2,162,250	2,218,363	2,297,550
C03	390,691	360,336	408,291	453,910	344,257	404,216	308,696	393,698	372,724	392,674	378,356
C04	1,100,274	1,094,411	1,238,286	1,486,415	1,847,711	1,811,061	1,810,762	1,813,366	1,709,356	1,899,976	2,129,268
C05	464,182	478,914	433,327	469,695	594,760	613,958	614,761	425,032	513,281	646,796	472,081
C06	661,100	559,579	605,081	688,001	715,972	907,672	1,293,443	1,259,274	894,763	1,049,817	1,183,657
C07	750,646	754,143	595,570	658,110	752,205	749,969	885,844	1,040,553	1,165,573	1,141,750	1,100,185
C08	322,443	320,122	306,442	345,764	358,594	254,427	377,587	423,274	306,052	423,173	260,336
C09	1,724,992	1,525,022	1,887,910	2,095,574	1,789,929	2,642,674	2,793,268	2,732,097	2,426,980	2,794,684	2,925,209
C10	946,677	791,422	710,994	845,434	895,225	923,498	1,115,124	843,032	810,161	937,407	918,195
C11	945,789	705,284	732,372	881,454	993,150	1,075,521	1,044,284	901,874	866,219	1,222,245	828,817
C12	240,400	238,902	332,778	369,358	432,711	563,594	486,235	472,602	425,896	365,770	433,298
C13	957,648	1,068,237	946,638	942,160	941,624	1,040,052	1,122,140	956,581	933,715	692,871	980,270
C14	233,785	269,280	209,551	358,862	329,225	360,648	380,023	263,913	283,484	309,455	364,091
C15	2,835,270	2,661,788	2,839,100	3,179,298	3,706,195	4,055,197	4,689,486	4,185,686	4,072,240	3,822,063	4,589,194
C16	11,007,182	10,457,110	11,257,882	12,250,437	12,980,634	13,518,348	13,883,318	14,777,153	13,712,972	14,377,967	15,428,448
C17	706,518	813,155	782,687	816,298	1,003,055	1,178,670	1,301,433	1,138,693	1,185,770	1,229,870	1,335,557
C18	18,722,979	20,161,584	20,457,563	24,009,936	25,449,834	28,191,719	28,538,278	31,700,666	31,267,048	32,630,282	33,661,409
C19	3,320,265	3,615,571	4,080,230	4,318,102	4,827,595	4,937,361	5,571,822	5,724,178	5,754,906	5,938,581	6,442,636
C20	13,325,027	13,627,239	14,036,588	15,271,883	16,930,381	18,332,079	18,565,203	20,611,984	18,700,173	20,788,847	21,476,346
C21	688,125	909,366	857,875	994,302	798,343	1,113,690	1,155,465	1,207,463	1,059,434	1,360,035	1,347,593
C22	2,033,398	2,031,538	2,165,984	2,113,543	2,536,138	2,860,143	3,028,005	2,887,632	2,820,931	3,139,322	3,696,074
C23	1,034,689	913,728	1,170,088	1,084,060	1,276,410	1,078,872	1,096,610	1,043,965	1,111,267	949,171	1,067,309
C24	760,891	709,726	788,946	996,653	1,203,597	1,280,329	1,259,046	1,398,779	1,499,860	1,385,415	1,436,303
C25	5,867,378	5,270,989	6,555,745	6,829,551	7,302,997	7,941,241	8,799,875	9,099,304	9,244,720	9,338,887	9,374,420
C26	597,209	586,150	563,057	605,297	721,446	649,986	433,226	270,635	334,493	312,941	227,348
C30	323,976	370,171	354,481	424,858	440,879	415,202	461,582	372,704	420,126	492,994	515,879
C31	504,921	583,929	591,425	500,174	436,534	686,479	623,141	619,864	735,761	585,580	660,535
C32	6,267,910	5,452,466	6,144,365	5,924,670	6,211,835	6,475,654	6,663,657	6,144,350	5,953,788	6,329,403	4,964,095
C33	68,167	132,538	97,623	111,840	108,786	98,122	60,024	75,285	56,573	57,077	86,258
C34	35,429,097	34,786,548	36,421,484	39,017,752	38,950,938	39,661,056	40,255,561	39,637,596	34,930,806	34,728,501	36,020,812
C37	193,692	144,356	154,034	244,083	371,627	275,280	352,447	401,454	411,470	488,964	421,374
C38	428,525	499,774	595,733	508,234	583,291	648,589	515,857	639,823	517,196	446,483	665,884
C39	147,306	199,878	219,955	164,318	249,808	179,952	233,763	143,539	156,839	120,907	149,671
C40	1,049,694	1,075,455	1,038,326	1,390,948	1,381,460	1,630,178	1,333,102	1,407,259	1,095,088	1,247,952	1,253,199
C41	838,174	727,968	646,048	990,272	1,100,546	1,125,885	1,295,392	1,293,814	1,198,548	1,198,718	1,496,595
C43	6,245,000	6,412,832	6,698,643	7,597,486	7,960,231	9,453,562	9,045,526	10,199,734	10,826,090	10,991,604	11,164,673
C44	3,256,454	2,858,097	3,339,161	3,501,216	3,254,021	3,828,605	4,400,571	5,029,132	4,162,406	4,616,343	5,478,748

C45	486,931	504,137	537,290	488,923	464,078	509,599	570,327	521,828	685,164	731,103	790,925
C46	41,223	54,887	85,592	54,912	46,399	104,173	82,046	85,730	132,932	65,464	98,295
C47	74,863	78,206	98,191	81,361	68,990	109,107	104,653	55,844	91,094	82,565	109,662
C48	701,678	1,033,496	682,301	824,107	1,029,470	1,113,224	1,055,977	1,301,673	1,264,396	1,287,600	1,192,239
C49	3,073,331	3,034,101	3,299,413	3,693,830	3,886,839	4,046,353	4,413,628	5,279,084	5,125,800	5,362,805	5,411,770
C50	76,012,841	77,293,860	83,007,170	90,229,432	95,548,489	105,117,579	114,335,036	131,138,610	135,768,976	135,847,986	146,383,472
C51	692,482	561,959	731,153	731,411	719,273	718,783	858,755	778,398	973,389	755,938	939,147
C52	232,010	198,530	251,006	219,062	237,817	230,223	279,658	286,871	261,535	206,739	351,609
C53	11,147,711	10,074,249	10,715,878	10,616,497	10,755,387	11,608,234	11,115,288	11,099,023	10,642,150	11,634,042	12,035,616
C54	9,995,323	10,659,130	11,507,263	12,568,828	13,647,773	14,439,146	15,421,925	17,077,807	16,178,080	16,848,164	17,911,777
C55	227,735	213,758	200,610	202,773	244,160	211,231	196,345	136,713	177,963	216,325	159,143
C56	14,938,854	14,976,891	15,872,720	15,951,488	17,523,317	18,425,360	19,857,769	22,137,652	21,546,247	20,893,306	21,113,146
C57	361,649	398,408	393,741	448,200	430,017	384,108	514,103	502,593	509,468	513,689	576,839
C58	119,797	166,486	121,114	157,097	190,375	121,861	94,713	204,346	190,329	146,286	128,608
C60	582,446	631,043	804,146	709,412	820,587	893,056	1,124,771	937,656	1,003,273	1,018,229	1,115,787
C61	14,743,226	16,978,448	20,195,665	25,011,459	29,080,609	34,960,156	37,821,367	42,291,202	44,527,022	41,305,817	50,148,585
C62	6,909,408	6,979,153	6,860,724	7,279,262	7,280,318	8,646,619	9,159,728	9,564,875	9,320,048	9,063,525	8,898,216
C63	137,464	134,521	164,113	100,589	111,566	140,573	88,477	134,232	93,567	78,317	116,571
C64	11,447,647	12,307,330	11,889,299	13,428,704	13,520,393	14,183,136	15,511,181	16,142,840	14,834,234	15,463,839	14,998,493
C65	578,332	538,481	589,393	619,487	655,496	723,065	743,871	695,770	599,326	684,484	837,844
C66	261,859	241,599	239,708	305,713	277,439	350,780	381,972	364,742	402,298	286,472	355,287
C67	11,564,458	11,590,942	12,122,748	13,280,927	14,227,154	15,759,691	16,988,497	17,318,038	17,668,047	17,123,853	17,082,738
C68	122,943	129,603	129,730	52,561	115,129	102,125	99,001	109,309	128,088	89,536	72,662
C69	1,247,661	1,275,412	1,508,475	1,443,762	1,652,382	1,907,320	1,884,135	2,218,440	2,018,293	1,853,029	2,302,788
C70	306,309	314,808	279,293	407,310	388,919	474,317	346,698	382,115	180,437	261,528	360,525
C71	10,779,447	10,788,099	11,743,394	13,033,317	12,851,081	14,793,836	14,501,197	14,493,074	15,300,834	15,476,692	16,409,833
C72	619,071	675,144	642,065	671,880	573,733	646,541	811,106	709,731	556,767	580,243	748,910
C73	9,977,656	10,489,471	11,578,305	14,966,254	17,231,541	18,639,477	19,967,586	22,311,388	21,250,810	21,600,554	22,506,766
C74	317,360	339,158	350,092	416,378	259,887	488,467	488,476	558,643	402,504	266,975	408,780
C75	259,519	296,010	228,409	288,584	505,090	486,326	431,375	609,316	943,608	678,493	1,094,390
C76	832,123	784,442	806,585	994,806	745,949	919,774	884,967	961,027	1,002,551	942,418	923,322
C77	647,709	684,344	732,209	709,748	758,634	1,115,458	1,167,548	1,098,361	1,105,187	1,333,676	1,164,823
C78	1,738,706	1,675,089	1,981,387	2,330,086	2,828,609	3,012,725	3,302,596	2,983,704	3,073,192	3,129,955	3,185,099
C79	1,954,986	1,882,819	2,066,817	2,100,192	2,547,173	2,876,248	2,998,870	3,286,501	3,101,839	3,268,834	3,327,748
C80	2,417,474	2,427,883	2,743,184	2,504,059	2,260,958	2,243,950	2,634,925	2,407,894	2,633,281	2,462,354	2,133,169
C81	4,960,391	4,771,136	5,121,889	5,379,828	5,827,261	6,025,356	6,301,271	6,341,767	6,971,487	7,066,389	6,984,816
C82	1,668,038	1,748,853	2,040,887	1,887,679	2,020,795	2,114,363	2,540,114	2,475,010	3,377,183	3,261,645	3,383,694
C83	3,692,321	3,782,533	3,854,910	3,815,242	4,183,742	4,407,187	3,760,768	3,410,494	3,731,873	3,784,593	4,497,475
C84	862,133	859,396	846,739	985,654	922,943	1,220,935	916,246	821,211	1,133,834	930,327	986,400
C85	2,604,308	3,144,034	3,673,158	4,336,910	4,742,182	5,732,667	6,241,929	6,591,616	7,539,074	6,598,666	6,743,649
C88	184,979	150,226	168,584	211,841	256,672	281,239	323,799	432,271	273,283	380,692	409,449
C90	3,250,565	3,337,090	4,291,407	4,488,549	4,837,066	5,610,900	6,071,601	5,701,944	6,012,628	5,985,964	5,965,874
C91	5,309,294	4,986,163	5,841,906	6,169,761	6,848,737	6,847,660	7,331,914	7,289,247	8,859,115	7,066,933	7,030,843

C92	3,901,501	4,392,875	4,804,474	4,508,953	4,393,667	5,517,805	5,784,927	3,963,760	4,861,173	4,182,277	4,755,024
C93	147,225	133,173	151,677	165,661	180,557	216,165	152,107	95,038	149,316	110,994	163,824
C94	220,797	246,040	228,572	246,099	292,297	224,358	188,453	155,328	171,677	215,235	117,463
C95	44,369	83,124	108,271	100,001	55,349	58,556	44,044	42,710	43,589	56,096	63,301
C96	415,941	375,089	466,572	622,593	433,927	504,572	401,850	290,283	378,082	226,890	255,209
C97	141,659	173,624	144,280	117,130	154,838	171,294	127,259	70,735	66,569	42,263	64,972
D00	89,222	83,441	106,076	115,535	143,368	166,453	105,237	123,787	53,585	68,840	136,186
D01	n.a.	n.a.	n.a.	n.a.	n.a.	n.a.	n.a.	n.a.	26,071	226,890	272,372
D02	101,565	89,945	114,936	119,565	125,729	129,681	120,925	60,911	50,287	78,535	120,472
D03	127,703	94,070	132,006	138,121	114,521	154,071	187,478	209,310	170,338	156,089	171,068
D04	167,151	168,151	155,822	152,815	199,846	199,967	199,366	176,838	115,516	147,375	157,472
D05	795,095	1,024,771	1,109,125	1,456,020	1,775,245	1,888,608	2,296,119	3,144,203	2,703,354	3,301,729	4,217,971
D06	384,640	400,946	365,536	349,290	490,580	464,355	627,136	877,779	845,816	922,158	985,620
D07	74,944	77,810	97,866	131,908	110,437	264,575	433,421	457,607	448,155	451,276	442,214
D09	252,743	206,779	256,615	243,496	330,355	343,053	435,857	596,286	711,029	820,640	759,832
D10	752,985	780,873	916,969	869,448	879,237	1,093,302	905,722	995,774	758,328	784,585	998,770
D11	1,433,124	1,712,130	1,744,118	2,242,260	2,397,723	3,236,524	3,159,941	3,387,846	3,053,716	3,012,643	3,720,926
D12	2,699,904	2,997,377	3,232,435	3,486,775	3,445,526	3,819,482	4,131,339	4,192,305	3,152,024	3,119,062	3,543,283
D13	1,594,547	1,697,615	1,730,869	1,920,090	1,807,567	1,940,648	1,999,019	1,693,303	1,403,819	1,350,559	1,912,508
D14	1,140,771	1,071,092	1,138,225	1,363,156	1,230,185	1,685,011	1,466,012	1,532,080	1,205,658	1,335,309	1,569,814
D15	830,187	649,841	583,784	770,370	590,329	805,268	764,724	755,647	600,253	791,666	796,720
D16	5,783,883	6,116,743	6,554,282	7,126,364	7,545,854	8,211,309	9,001,579	9,351,842	7,009,718	7,632,362	8,697,057
D17	4,463,941	4,556,743	5,052,635	5,562,114	6,483,974	6,832,672	7,319,928	8,043,033	5,011,211	6,745,061	9,211,153
D18	1,049,694	1,206,486	1,424,508	1,621,933	1,695,480	2,004,697	2,019,969	2,089,379	1,620,941	1,911,522	1,853,776
D19	33,156	44,417	53,729	47,692	48,745	74,289	38,197	35,574	39,055	21,131	22,066
D20	515,812	483,197	448,121	510,669	622,391	603,346	544,115	338,268	339,027	420,668	504,734
D21	9,460,634	9,972,565	10,386,270	10,937,155	12,696,330	13,908,786	14,506,751	16,080,895	10,998,901	13,636,734	16,571,762
D22	2,954,502	3,263,405	3,533,105	4,258,992	4,699,780	5,092,642	5,428,485	6,295,024	4,173,639	5,052,805	6,655,496
D23	10,735,239	10,606,701	11,734,127	12,276,634	13,136,861	13,948,910	14,403,560	15,413,667	10,271,075	12,186,509	14,032,599
D24	4,565,345	3,710,117	3,568,464	3,285,177	3,515,125	3,484,435	3,250,465	3,517,631	2,663,887	2,833,570	2,946,160
D25	77,657,485	76,571,602	78,205,622	78,385,214	79,048,646	83,095,141	90,569,946	98,784,264	74,885,808	89,958,504	103,504,780
D26	1,096,967	1,318,005	1,540,095	1,770,214	1,886,463	1,828,656	1,790,884	1,438,594	1,479,868	1,359,491	1,501,276
D27	34,284,292	34,157,091	35,486,633	37,102,532	39,000,117	40,685,841	42,953,628	45,144,087	36,718,994	39,450,500	40,002,073
D28	889,158	931,416	1,016,379	1,009,668	950,487	901,248	865,771	884,915	730,917	638,299	649,167
D29	351,162	266,028	241,984	289,424	297,163	317,173	296,516	225,133	230,106	186,697	223,336
D30	1,009,519	794,991	832,108	872,470	976,728	976,375	886,818	769,504	654,971	734,916	832,829
D31	349,790	392,380	282,788	238,962	337,219	270,253	309,768	447,887	449,391	524,473	400,979
D32	3,021,217	2,986,035	3,394,353	3,602,057	4,491,418	4,858,323	5,243,248	5,470,400	4,938,665	5,209,112	6,234,791
D33	6,492,983	6,366,670	6,624,349	7,043,911	7,494,675	8,072,691	8,882,408	8,684,614	8,384,272	7,957,938	8,811,957
D34	1,416,667	1,252,093	1,532,291	1,362,149	1,190,303	1,209,577	1,227,962	1,154,412	891,054	1,084,238	981,831
D35	2,974,912	3,429,494	3,285,513	3,678,968	3,850,432	4,542,919	4,872,384	5,250,024	4,532,554	5,576,951	6,080,997
D36	540,336	729,079	708,312	719,572	715,885	933,831	783,627	659,782	666,719	758,117	987,514
D37	10,489,031	11,166,043	12,784,321	15,021,922	16,897,624	21,017,763	23,921,971	27,050,340	25,650,432	28,365,657	30,616,286

D38	12,444,662	13,557,123	15,263,900	15,413,614	16,774,501	18,996,215	20,992,285	22,663,823	20,306,686	23,694,852	24,742,911
D39	951,597	948,627	1,139,688	1,372,140	1,597,816	2,156,814	3,447,102	4,028,704	4,316,669	5,462,362	5,950,607
D40	1,120,120	1,311,977	1,727,374	2,071,897	2,483,222	3,065,602	3,921,254	4,751,775	4,499,063	5,303,005	5,880,508
D41	3,930,946	4,517,085	5,696,326	7,542,657	9,059,560	10,734,822	12,192,994	14,595,041	12,860,252	14,873,249	16,326,695
D42	388,512	374,454	535,257	553,743	730,308	737,867	672,446	733,103	413,221	506,936	475,982
D43	5,531,947	6,528,000	6,908,113	7,504,118	7,629,094	8,384,651	10,010,102	10,007,901	9,371,056	10,916,881	10,905,786
D44	2,088,497	2,233,002	2,920,953	3,996,688	4,801,875	5,873,892	6,363,829	7,380,665	6,564,140	7,284,565	8,022,258
D45	482,010	459,244	621,500	728,304	792,174	846,602	827,963	913,353	1,281,604	805,935	1,071,544
D46	836,076	926,339	1,016,542	911,933	1,179,702	1,466,518	1,434,734	1,381,613	1,336,426	1,492,379	1,333,885
D47	1,473,943	1,573,881	2,011,706	2,167,448	2,547,434	3,287,447	3,822,546	4,215,573	4,932,585	4,935,166	4,786,229
D48	14,251,293	15,088,411	17,366,401	19,179,905	22,339,094	25,288,008	27,429,585	30,832,401	25,854,260	29,931,560	33,155,560

Notes: n.a. – not available. The names of diseases for each ICD-10 code are available in the World Health Organization website: https://icd.who.int/browse10/2019/en#/II.

Supplementary table 5. Neoplasm-related work absence per episode productivity losses by single ICD-10 diagnoses in Poland in the years 2012-2022

	Per absence episode losses (€)										
	2012	2013	2014	2015	2016	2017	2018	2019	2020	2021	2022
C00	1,748	1,539	1,811	2,137	2,019	1,945	2,111	2,278	2,111	2,072	2,394
C01	2,198	1,987	2,245	2,184	2,413	2,332	2,771	2,458	2,481	2,728	2,726
C02	2,112	1,920	1,990	2,043	2,216	2,438	2,383	2,383	2,278	2,625	2,536
C03	1,852	1,917	1,917	1,923	1,990	2,001	2,114	2,250	2,405	2,600	2,646
C04	2,205	2,257	2,199	2,259	2,438	2,636	2,879	2,594	2,598	2,687	2,772
C05	1,950	2,029	1,943	2,116	2,102	2,095	2,720	2,249	2,222	2,310	2,098
C06	1,873	2,057	1,997	2,048	2,340	2,453	2,746	2,549	2,425	2,706	2,721
C07	2,029	1,881	1,731	1,823	1,959	2,016	2,161	2,073	2,259	2,321	2,120
C08	1,671	1,423	1,621	1,631	1,552	1,621	1,917	2,263	1,866	2,489	2,304
C09	2,478	2,290	2,302	2,376	2,346	2,583	2,699	2,653	2,644	2,795	2,797
C10	2,332	2,077	2,222	2,162	2,313	2,476	2,564	2,502	2,433	2,765	2,654
C11	2,225	1,948	2,267	2,031	2,188	2,204	2,363	2,392	2,533	2,734	2,717
C12	2,557	2,192	2,295	2,225	2,688	2,736	2,657	2,748	2,662	2,750	2,708
C13	2,152	2,027	2,018	2,166	2,190	2,436	2,737	2,537	2,779	2,625	2,850
C14	1,635	1,662	1,977	2,051	1,819	2,147	2,262	2,399	2,198	2,691	2,890
C15	2,037	2,150	2,138	2,258	2,403	2,506	2,591	2,532	2,512	2,651	2,693
C16	2,168	2,103	2,181	2,230	2,285	2,396	2,458	2,499	2,523	2,573	2,433
C17	1,835	1,857	1,791	1,930	1,998	1,968	2,082	2,063	2,229	2,146	1,961
C18	1,954	1,933	1,949	1,994	2,038	2,204	2,311	2,342	2,362	2,366	2,314
C19	1,999	1,938	2,048	2,012	2,166	2,233	2,341	2,393	2,412	2,354	2,399
C20	2,107	2,063	2,055	2,104	2,181	2,355	2,442	2,488	2,434	2,514	2,524
C21	1,949	1,931	2,014	2,021	2,255	2,380	2,464	2,474	2,397	2,736	2,855
C22	1,845	1,751	1,918	1,994	2,160	2,226	2,362	2,350	2,347	2,494	2,417
C23	1,923	1,778	1,924	1,841	2,023	2,087	2,374	2,341	2,602	2,538	2,377
C24	2,162	2,011	1,967	2,034	2,284	2,231	2,493	2,539	2,608	2,344	2,426
C25	2,076	1,983	2,187	2,064	2,177	2,333	2,371	2,313	2,423	2,497	2,440
C26	1,838	1,804	2,062	2,018	2,050	2,145	2,166	2,416	2,356	2,371	2,773
C30	1,291	1,429	1,244	1,501	1,495	1,597	1,931	2,118	2,090	2,370	2,433
C31	2,359	2,093	2,240	2,203	2,109	2,552	2,388	2,431	2,366	2,603	2,718
C32	2,282	2,186	2,233	2,257	2,366	2,446	2,548	2,503	2,584	2,634	2,650
C33	1,794	1,949	1,877	2,237	2,417	2,582	2,309	2,035	2,460	1,359	1,875
C34	2,105	2,081	2,157	2,225	2,302	2,496	2,620	2,658	2,661	2,791	2,734
C37	1,881	1,977	1,621	2,141	2,173	1,823	2,319	2,750	2,494	2,495	2,601
C38	1,808	1,872	1,922	1,993	2,061	2,420	2,659	2,677	2,523	2,626	2,870
C39	1,754	1,574	1,666	1,694	2,082	1,836	1,998	2,208	2,178	2,572	2,537
C40	1,898	1,973	1,812	1,890	1,991	2,191	2,182	2,665	2,601	2,737	2,874
C41	2,085	2,261	2,019	2,341	2,357	2,662	2,822	2,588	2,663	2,534	2,766
C43	1,638	1,631	1,599	1,672	1,673	1,724	1,738	1,672	1,798	1,708	1,599

C44	1,277	1,219	1,285	1,284	1,223	1,365	1,441	1,472	1,469	1,431	1,410
C45	1,996	1,954	2,035	2,054	2,100	2,275	2,437	2,635	2,697	2,698	2,756
C46	1,874	1,715	1,861	1,569	1,856	1,488	1,415	1,453	1,602	1,148	1,755
C47	1,528	1,348	2,395	1,627	1,500	1,581	1,716	2,327	2,118	2,293	3,046
C48	1,944	1,914	1,815	1,852	1,991	2,020	1,956	2,069	2,199	2,164	2,028
C49	1,923	1,890	1,897	1,938	1,947	2,087	2,100	2,136	2,224	2,273	2,153
C50	1,993	1,938	1,962	2,009	2,051	2,171	2,265	2,293	2,423	2,375	2,232
C51	2,025	1,899	1,810	2,066	2,049	2,139	2,359	2,303	2,427	2,362	2,402
C52	1,813	1,805	1,832	1,752	1,949	1,968	2,311	2,706	2,444	2,685	2,442
C53	2,153	2,091	2,206	2,157	2,211	2,322	2,439	2,472	2,526	2,621	2,550
C54	2,060	2,017	2,037	2,064	2,119	2,243	2,407	2,520	2,535	2,574	2,577
C55	1,423	1,595	1,508	1,459	1,420	1,542	1,785	1,799	2,046	2,301	2,306
C56	1,854	1,852	1,877	1,928	2,015	2,098	2,256	2,291	2,338	2,400	2,318
C57	1,674	1,836	1,823	1,837	2,009	2,246	2,107	2,244	2,359	2,423	2,508
C58	1,664	1,411	1,553	1,390	1,523	1,934	2,059	1,675	1,866	1,573	1,588
C60	1,885	1,778	2,000	1,801	1,949	2,189	2,353	2,386	2,195	2,407	2,394
C61	1,984	2,015	2,051	2,100	2,161	2,357	2,432	2,522	2,541	2,636	2,643
C62	1,737	1,633	1,635	1,761	1,812	1,975	2,059	2,040	2,096	2,127	2,056
C63	1,656	1,387	1,609	1,734	1,528	1,926	1,609	2,131	2,176	2,303	1,943
C64	2,021	1,991	1,985	2,105	2,185	2,271	2,441	2,470	2,505	2,589	2,500
C65	1,830	1,844	1,998	1,967	2,068	2,274	2,339	2,312	2,508	2,507	2,756
C66	1,857	1,817	1,918	1,698	1,954	2,063	2,372	2,224	2,514	2,292	2,450
C67	1,539	1,499	1,541	1,625	1,662	1,800	1,852	1,846	1,959	1,973	1,897
C68	1,518	1,683	1,544	1,502	1,622	1,824	1,941	2,102	2,372	2,558	2,018
C69	1,772	1,665	1,704	1,663	1,796	1,870	1,913	2,111	2,107	2,030	2,078
C70	1,791	1,943	1,814	1,997	1,984	2,248	2,442	2,547	2,123	2,539	2,955
C71	2,382	2,370	2,411	2,521	2,632	2,906	3,018	2,971	2,992	3,106	3,214
C72	2,010	1,929	1,877	2,024	2,049	2,237	2,600	2,499	2,554	2,844	3,069
C73	1,535	1,506	1,561	1,632	1,687	1,776	1,832	1,900	1,907	1,924	1,885
C74	1,644	1,535	1,563	1,735	1,397	1,886	1,931	1,868	2,023	1,907	1,817
C75	1,260	1,213	1,352	1,306	1,619	1,406	1,331	1,398	1,757	1,336	1,537
C76	1,913	1,877	1,982	2,022	2,101	2,134	2,164	2,023	2,315	2,255	2,123
C77	1,861	1,906	1,953	1,913	1,955	2,081	2,402	2,260	2,171	2,390	2,210
C78	2,184	2,078	2,144	2,291	2,397	2,408	2,592	2,546	2,503	2,530	2,536
C79	2,232	2,192	2,208	2,273	2,403	2,514	2,659	2,773	2,812	2,937	2,815
C80	1,908	1,931	2,037	2,094	2,141	2,390	2,486	2,513	2,475	2,614	2,656
C81	1,701	1,571	1,660	1,707	1,792	1,937	2,006	2,055	2,146	2,101	1,980
C82	1,619	1,530	1,624	1,668	1,676	1,657	1,930	1,964	2,054	2,099	1,831
C83	1,865	1,847	1,935	2,006	2,127	2,210	2,293	2,210	2,372	2,409	2,464
C84	1,735	1,779	1,790	1,832	1,789	1,976	2,014	1,841	2,096	2,100	2,038
C85	1,867	1,859	1,909	2,069	2,110	2,287	2,312	2,271	2,257	2,268	2,255
C88	1,412	1,502	1,492	1,891	1,746	1,981	2,103	2,217	1,822	2,163	1,728
C90	1,904	1,814	1,992	2,062	2,048	2,291	2,381	2,133	2,342	2,334	2,290

C91	1,813	1,763	1,846	1,897	1,949	2,043	2,132	2,083	2,107	2,178	1,985
C92	1,820	1,872	1,856	1,921	1,919	2,125	2,026	1,614	1,812	1,889	1,805
C93	2,103	1,958	1,920	1,841	1,753	2,099	2,414	2,263	2,620	2,643	2,445
C94	1,338	1,224	1,329	1,309	1,419	1,079	1,356	1,726	2,020	1,736	1,468
C95	1,056	1,319	1,388	1,587	1,419	1,428	1,190	1,256	1,406	1,810	1,918
C96	1,582	1,374	1,481	1,572	1,409	1,577	1,667	1,640	1,703	1,621	1,519
C97	1,460	1,536	1,551	1,673	1,720	1,903	1,899	1,965	2,147	2,013	2,406
D00	1,026	869	1,071	1,090	1,225	1,095	1,169	1,719	1,307	1,405	1,661
D01	n.a.	n.a.	n.a.	n.a.	n.a.	n.a.	n.a.	n.a.	2,173	1,773	1,522
D02	883	789	1,017	873	898	1,072	1,152	1,692	1,524	1,671	2,459
D03	1,150	1,022	1,031	973	1,060	1,070	1,349	1,434	1,310	1,394	1,205
D04	908	837	824	955	1,020	1,075	1,114	1,134	1,080	1,170	1,029
D05	1,725	1,982	1,766	1,822	1,871	1,957	2,152	2,241	2,351	2,353	2,267
D06	1,526	1,293	1,445	1,275	1,418	1,420	1,455	1,656	1,685	1,683	1,699
D07	1,190	1,389	1,461	1,649	1,578	2,242	2,234	2,254	1,948	2,338	2,303
D09	1,708	1,567	1,594	1,485	1,430	1,759	1,879	2,107	2,161	2,110	2,215
D10	868	856	902	865	903	981	923	942	1,096	980	1,022
D11	1,352	1,304	1,342	1,420	1,465	1,576	1,671	1,679	1,682	1,773	1,766
D12	901	907	909	909	905	932	923	879	921	933	941
D13	1,283	1,338	1,360	1,377	1,323	1,397	1,442	1,348	1,528	1,504	1,514
D14	1,482	1,479	1,454	1,619	1,528	1,714	1,672	1,857	1,835	1,905	1,853
D15	1,953	1,679	1,640	1,716	1,752	2,044	2,089	2,123	2,129	2,399	2,142
D16	1,777	1,813	1,890	1,922	1,995	2,128	2,252	2,317	2,286	2,346	2,387
D17	858	841	866	895	952	1,002	1,067	1,094	1,105	1,119	1,148
D18	1,363	1,350	1,344	1,404	1,420	1,433	1,492	1,552	1,597	1,655	1,531
D19	829	854	995	1,109	1,016	1,376	1,005	1,227	1,220	1,761	1,697
D20	1,379	1,469	1,392	1,426	1,564	1,671	1,838	1,944	2,006	2,274	2,315
D21	1,014	981	1,014	1,061	1,139	1,234	1,284	1,339	1,333	1,344	1,327
D22	574	550	560	578	603	641	644	646	673	642	611
D23	701	678	692	709	740	775	804	808	853	851	813
D24	903	863	849	839	833	860	843	855	951	918	869
D25	2,067	2,012	2,040	2,090	2,119	2,241	2,296	2,353	2,306	2,364	2,371
D26	1,532	1,488	1,519	1,510	1,623	1,805	1,763	1,544	1,633	1,534	1,655
D27	1,795	1,753	1,805	1,841	1,873	1,995	2,056	2,095	2,076	2,168	2,156
D28	1,244	1,285	1,285	1,291	1,350	1,433	1,445	1,651	1,869	1,720	1,595
D29	1,345	1,243	1,228	1,322	1,259	1,422	1,386	1,511	1,704	1,518	1,509
D30	1,318	1,166	1,206	1,340	1,382	1,328	1,447	1,417	1,541	1,537	1,583
D31	980	1,019	1,150	1,171	1,282	1,140	1,095	1,111	1,221	1,276	1,093
D32	2,125	2,093	2,071	2,188	2,262	2,386	2,390	2,452	2,411	2,666	2,741
D33	2,119	2,143	2,094	2,163	2,181	2,379	2,580	2,501	2,573	2,751	2,775
D34	1,876	1,950	1,923	1,900	1,860	1,911	1,872	1,997	2,044	2,012	1,925
D35	1,364	1,328	1,304	1,275	1,288	1,383	1,383	1,362	1,471	1,516	1,420
D36	1,280	1,386	1,300	1,340	1,393	1,613	1,513	1,575	1,759	1,634	1,679

D37	1,391	1,358	1,355	1,372	1,420	1,514	1,580	1,572	1,673	1,723	1,656
D38	1,582	1,576	1,624	1,633	1,682	1,777	1,831	1,866	1,877	1,924	1,914
D39	1,217	1,183	1,201	1,228	1,295	1,362	1,598	1,632	1,714	1,809	1,850
D40	1,337	1,351	1,363	1,326	1,301	1,419	1,526	1,495	1,526	1,573	1,518
D41	1,496	1,469	1,501	1,590	1,656	1,773	1,823	1,877	1,922	2,011	2,025
D42	1,807	1,750	1,968	1,992	2,161	2,177	2,044	2,357	2,056	2,325	2,288
D43	2,096	2,106	2,157	2,157	2,294	2,425	2,531	2,577	2,565	2,827	2,757
D44	1,620	1,611	1,657	1,783	1,867	1,875	1,913	1,965	1,966	2,010	1,989
D45	920	930	922	982	991	957	1,032	1,025	1,312	1,012	1,051
D46	1,717	1,675	1,566	1,628	1,627	1,878	1,839	1,742	1,960	2,218	2,040
D47	1,251	1,235	1,299	1,286	1,291	1,437	1,394	1,341	1,504	1,490	1,360
D48	1,113	1,090	1,095	1,105	1,162	1,226	1,230	1,219	1,305	1,281	1,189

Notes: n.a. – not available. The names of diseases for each ICD-10 code are available in the World Health Organization website: https://icd.who.int/browse10/2019/en#/II.

Supplementary table 6. Comparison of productivity losses associated with mortality and short-term work absence by cancer site in Poland (2018 data)

	Total losses				Per death / per absence episode losses			
	Mortality losses[a]		Work absence losses		Mortality losses[a]		Work absence losses	
	thousand €	rank	thousand €	rank	€	rank	€	rank
oral cavity and pharynx (C00-C14)	113,000	5	15,420	11	59,384	6	2,545	5
oesophagus (C15)	41,400	14	4,689	21	52,953	10	2,591	3
stomach (C16)	97,000	6	13,883	12	55,029	7	2,458	6
colorectum (C18-C21)	147,000	2	53,831	2	42,515	17	2,361	15
liver (C22)	36,800	16	3,028	22	54,081	8	2,362	14
gallbladder (C23-C24)	19,400	18	2,356	23	39,506	19	2,436	9
pancreas (C25)	81,800	7	8,800	17	44,983	16	2,371	12
larynx (C32)	41,300	15	6,664	18	48,064	14	2,548	4
lung (C33-C34)	291,000	1	40,316	3	31,511	21	2,619	2
melanoma skin (C43)	47,800	12	9,046	16	79,872	4	1,738	23
breast (C50)	139,000	3	114,335	1	52,114	11	2,265	16
cervix uteri (C53)	48,200	11	11,115	15	53,196	9	2,439	7
corpus uteri (C54)	9,999	22	15,422	10	25,640	23	2,407	11
ovary (C56)	67,100	8	19,858	6	48,850	13	2,256	17
prostate (C61)	18,800	19	37,821	4	26,956	22	2,432	10
kidney (C64-65)	42,700	13	16,255	8	47,306	15	2,436	8
bladder (C67)	32,700	17	16,988	7	34,745	20	1,852	21
brain and central nervous system (C70-72)	133,000	4	15,659	9	83,094	2	2,978	1
thyroid (C73)	4,986	23	19,968	5	50,364	12	1,832	22
Hodgkin lymphoma (C81)	12,100	21	6,301	20	130,012	1	2,006	20
non-Hodgkin lymphoma (C82-C85, C96)	53,300	10	13,861	13	80,133	3	2,182	18
multiple myeloma (C88, C90)	16,200	20	6,395	19	41,315	18	2,365	13
leukaemia (C91-C95)	60,600	9	13,501	14	71,219	5	2,067	19
correlation coefficient[b]	0.494				-0.234			

Notes: a – Mortality losses refer to unpublished estimates by M. Ortega-Ortega based on [11]; b – Pearson's correlation coefficient is calculated for monetary values, not ranks.
